# Solvent-Based Recycling as a Waste Management Strategy for Fibre-Reinforced Polymers: Current State of the Art

**DOI:** 10.3390/polym17070843

**Published:** 2025-03-21

**Authors:** Matthew J. Keith, Bushra Al-Duri, Tom O. McDonald, Gary A. Leeke

**Affiliations:** 1 School of Chemical Engineering, University of Birmingham, Birmingham B15 2TT, UK; b.al-duri@bham.ac.uk (B.A.-D.); g.a.leeke@bham.ac.uk (G.A.L.); 2 Department of Materials, The University of Manchester, Oxford Road, Manchester M13 9PL, UK; thomas.mcdonald@manchester.ac.uk; 3 Henry Royce Institute, The University of Manchester, Oxford Road, Manchester M13 9PL, UK

**Keywords:** composites, sustainable materials, fibre-reinforced polymers, carbon fibre, glass fibre, recycling, solvolysis, waste management

## Abstract

The growing use of fibre-reinforced polymers (FRPs) is driving a demand for the development of sustainable end-of-life strategies. Solvolysis, a chemical recycling method using solvents to decompose the polymer matrix, has emerged as a promising approach for reclaiming both fibres and organic compounds from FRP waste. This work provides a comprehensive overview of solvolysis techniques by discussing the environmental benefits and economic opportunities of this technology, summarising the process conditions, and evaluating the characteristics of the recovered products. The economic viability of solvolysis lies in recovering high-value components; predominantly carbon fibres from CFRPs and organic products from GFRPs, which are suitable for reuse or as a feedstock for new composites. Solvolysis can operate under low temperature and pressure (LTP) or high temperature and pressure (HTP) conditions. The choice of solvent, catalyst, reaction time, and temperature is crucial to achieving high resin decomposition while preserving fibre properties. To achieve an economically viable and environmentally beneficial process, it will be essential to optimise these parameters. A key challenge is maintaining the strength and surface properties of the recovered fibres, as degradation in their performance can limit their suitability for high-performance applications. The implication of this is that, without careful consideration of the recycling process, FRPs cannot be fully circular. They will be continuously downgraded into low-value applications and ultimately incinerated or landfilled. This review further explores the diversity of organic products obtained, which can range from monomers to oligomers to complex mixtures. Efficient separation and upgrading techniques, such as distillation and liquid–liquid extraction, are essential to maximise the value of the recovered organics. These additional processing steps are likely to result in greater financial and resource costs within a commercial recycling system. This review concludes with a summary of commercial solvent-based recycling ventures and an outlook on future research directions, which includes the need to develop processes capable of recovering high-value, long carbon fibres. Successful development of such a process would represent a step-change in the value proposition of a carbon fibre recycling industry.

## 1. Introduction

Due to their blend of desirable properties, composite materials have been used by humans for centuries [1]. They typically have high strength-to-weight ratios, exceptional stiffness, a wide operating temperature, and chemical stability, meaning that they find uses across a wide range of demanding applications. Composites tend to be fully synthetic and often consist of a fibre reinforcement bound together with a thermoplastic, or thermoset, matrix. This former category includes commonly used consumer plastics, such as polyethylene and polypropylene; however, so-called “engineering plastics”, which typically have high strength, stiffness and thermal durability, are often used in high-performance applications. These include polycarbonate (PC), polyamide (PA), polyphenylene sulfide (PPS), polyetherether ketone (PEEK) and polyetherimide (PEI) [2]. The chemical structure of these compounds consists of long, repeating units of the same monomer. By contrast, thermosetting resins contain smaller polymer chains (such as bisphenol A diglycidyl ether, or BADGE), and also undergo a curing stage whereby shorter organic compounds, such as amides, cross-link the polymer chains together. This results in a resin which does not melt, or even soften, when exposed to heat, although thermal degradation is possible at temperatures above 450 °C, depending on the material [3].

Globally, 90 to 95 wt.% of all polymeric composites are glass fibre-reinforced polymers (GFRPs), with the bulk of the remainder consisting of carbon fibre-reinforced polymers (CFRPs) [4]. Glass fibres are a much lower-value product than carbon fibres and hence, by market share, CFRPs contribute a disproportionate amount to the global economy. Estimates vary, but the value of the global CFRP market was in the region of USD 29.7 bn in 2022 and is expected to grow at a rate of up to 12.7% per annum until 2028 [5]. A lower growth rate of ~4% [6,7] has been predicted for GFRPs, leading to a market worth USD 76 bn by 2030 [6]. Due to this growth, the quantity and value of the waste being generated is expected to increase at similar rates, reaching an estimated 34,000 t/yr and 112,000 t/yr of CFRP and GFRP, respectively, by 2030 [8,9]. Currently, most composite waste is disposed of in landfill or incinerated [10], necessitating the manufacture of fully virgin components. To achieve net-zero and drive towards a circular composites industry, there is an urgent need to develop recycling processes which can recover high-quality materials for use in high-value applications. These processes may be categorised as mechanical, pyrolytic, solvolytic, or enzymatic, as briefly described in the following sections.

The most commercially mature technology in composites recycling relies on mechanical comminution techniques. For composites, these tend to use hammer mills, cutting mills or single/multi-shaft shredding machines. If composites are further ground into powders, the recyclate can be sieved to separate resin-rich fractions from fibre-rich portions [11]. As any fibres are likely to be less than 5 mm in length, their use as a reinforcement agent is severely limited and, as such, the fibre-rich portions may only find use as a filler material in a new composite. For thermoplastics, rather than thermosets, it is possible to use the resin-rich portion in the manufacture of new composites. However, due to the presence of fibres in the recyclate and resultant increase in viscosity, it is necessary to blend the recovered resin with virgin polymer [12]. This, and the loss of long, continuous fibres, means that the mechanical recycling of FRPs is not a fully closed loop.

Although some size reduction is necessary for large components (such as aircraft fuselage/wings or wind turbines), pyrolysis may represent an improvement in resource efficiency. In this process, the composite is heated in a controlled atmosphere to temperatures typically in the range of 450 to 700 °C. Long, continuous fibres can be recovered, and the resin is converted into a mixture of gases, condensable products such as oils or wax, and char. This latter component consists mostly of carbon and often contaminates the surface of the fibres. The most common method for removing it is an oxidation step, which subsequently generates CO_2_. Fortunately, at temperatures of more than 500 °C, there is little char formed on the fibre surface with most of the polymer being broken down into a mixture of different hydrocarbons. This may be recovered through cooling the exhaust stream, thus separating the mixture into a liquid fraction and a non-condensable gas. Pyrolysis is a mature FRP recycling technology with several companies (e.g., Gen 2 Carbon in the UK [13], Mitsubishi Chemical Advanced Materials in Germany [14], Carbon Rivers in the USA [15], and Toray/Toyota Tsusho in Japan [16]) all operating commercial processes. Unfortunately, glass fibres may suffer significantly under these conditions, with up to a 50% loss in mechanical performance when exposed to temperatures of 450 °C. The previous literature suggests that 500 to 550 °C is the upper temperature limit before the strength of carbon fibres is significantly affected [3]. Pyrolysis is also often associated with a high energy demand and the potential loss of the polymer matrix [17]. For these reasons, there has been significant academic research into alternative systems.

Enzymatic processes are exemplary of these alternative systems. These are highly selective, often targeting specific bonds such as ester, ether, or amide linkages. These bonds are subsequently cleaved, generating a mixture of monomers, oligomers and fibres [18]. As a biological system, they typically operate at a mild temperature, produce minimal harmful by-products, and can be tailored to specific FRP waste streams [18,19]. This mild temperature means these systems are energetically favourable, and there is minimal risk to the quality of the carbon fibres [20]. Micro-organisms such as *Rahnella* sp., *Stenotrophomonas* sp. and *Bacillus tropicus* are naturally occurring and generate enzymes which can break down common thermoplastics such as PET and PS, as well as thermoset epoxies [18]. To increase reaction kinetics, improve thermal stability, and provide selectivity towards specific products, directed molecular evolution has been applied to develop advanced ligninases for the recycling of thermosets. An example of this latter approach is in the recently concluded BIZENTE project [19]. To accelerate the biodegradability of polymer matrices, it is also possible to “design-in” specific chemical bonds which are later directly targeted by enzymes at that material’s end-of-life [21]. Although this represents a significant opportunity for future polymer systems, it does not address the current issue of FRP waste manufactured from conventional polymers. Moreover, existing enzymatic processes are slow, with reactions occurring over days or weeks, which therefore makes them inherently difficult to scale. There are also some concerns regarding their applicability to all materials; PEEK, for example, is biocompatible and hence whether it can be enzymatically degraded is currently uncertain [22]. With limited research successes, and no known commercial systems for the enzymatic recycling of FRPs, solvent-based processes have instead gained significant attention across academia and industry.

Commonly referred to as solvolysis, this approach to recycling FRPs offers a potential route to recovering both high-value fibres and organic compounds from the resin at a lower environmental cost than pyrolysis, and much more quickly than enzymatic processes. Where pyrolysis thermally degrades the resin, solvolysis aims to break specific chemical bonds to liberate clean fibres. The use of such a solvent facilitates lower temperatures than pyrolysis, avoids the generation of emissions due to the burning of the resin, and enables the potential recovery of organic compounds, which may be further used within the chemical industry. Although enzymatic processes facilitate even lower temperatures than solvolysis, this latter approach is potentially simpler to operate, two to three orders of magnitude faster, and at a higher technology readiness level. Solvolysis may be categorised based on the solvent; with hydrolysis, alcoholysis, and acetolysis, for example, referring to the use of water, alcohols, and acetone. Further, they may also be classified according to their reaction conditions. Generally, low temperature and pressure (LTP) conditions use temperatures of less than 200 °C and ambient pressure. Decomposition of the polymer using high temperatures and pressures (HTP) may use temperatures of up to 450 °C and a pressure of 0.3 to 30 MPa [23]. Due to the extreme heat, these systems are considered to be thermochemical recycling processes as they combine the solvent and high temperatures, which leads to the recovery of clean fibres.

**Figure 1:**
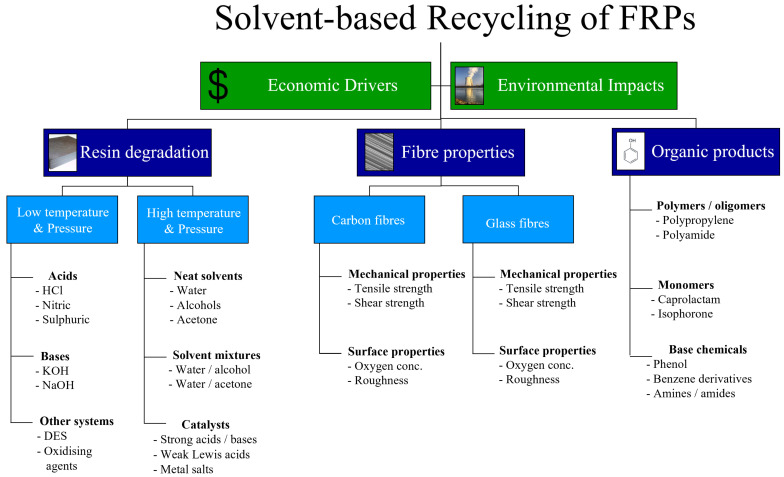
Considerations for the solvent-based recycling of fibre-reinforced polymers (FRPs), highlighting the need to consider economic drivers, environmental impacts, fibre quality and organic products.

This review article aims to provide a comprehensive insight into the current state of the art of solvolysis techniques for the recycling of FRPs. The primary considerations for solvent-based recycling techniques, which are discussed herein, are shown diagrammatically in Figure 1, which also reflects the structure of this article. The environmental and economic case for commercial uptake is presented, followed by a detailed discussion of various LTP and HTP processes. The properties of the fibres which can be recovered by these techniques are also considered, before discussing the organic products obtained and methods for their separation and upgrading. This paper concludes with a summary of currently commercialised systems, likely challenges, and an outlook for future solvolysis recycling processes.

## 2. Why Recycle Fibre-Reinforced Polymers?

### 2.1. Economic Case

In FRP recycling, the approach taken has usually been to recover the component of highest economic value. As such, this review reports the typical costs of virgin and recycled fibres and polymer matrix materials; the primary criteria for building an economic case for the recycling of FRPs. For CFRPs, this is the recovery and reuse of high-performance carbon fibres which can retail for ~USD 30/kg for low-strength material up to USD 85/kg for aerospace-grade tows [24]. Recycled carbon fibres are estimated to cost USD 18 to USD 25/kg [25] and so may be used as non-structural components within the aerospace and automotive industries where there is a need to balance price and performance. For comparison, common matrix materials (shown in Table 1) typically retail in the range of USD 1 to USD 20/kg [26]. In contrast, glass fibres may retail for as little as USD 0.75 to USD 3/kg depending on the grade [27,28], often making the resinous products the higher-value component. Recycling processes for GFRPs have hence mostly focused on recovering resin-rich fractions, organic monomers, or value-added chemicals, rather than high-quality fibres.

It has been estimated that, globally, nearly 80% of all FRPs use a thermoset resin as the matrix [40]. Therefore, there is a significant economic opportunity as, in a given mixture of composite waste, a high proportion is likely to consist of valuable resins. The downside is that recovering the polymer represents a major technical challenge as, when curing, chemical bonds are formed between a pre-polymer and a hardener. During solvolysis, it is usually different chemical bonds which are broken, so it is often not possible to recover the same monomers and hardener. However, different organic molecules have been identified, the approximate prices of which are provided in Table 2.

If these can be effectively separated and refined to a sufficient purity, it is hence possible that products obtained from the degradation of thermoset resins may represent an economic opportunity, alongside the recovered carbon fibres. In the selection of an FRP recycling process, the primary driver is likely to be the system’s economics and so it is necessary to balance financial cost with performance. For example, a cheaper reaction system may not be able to achieve sufficient degradation of high-performance polymers, and so a trade-off between total costs and achieving a high recovery yield must be considered.

The implementation of recent legislation also drives the economic case for recycling FRPs. According to the European Union’s Waste Framework Directive (Directive 2008/98/EC), landfilling and incineration are the least-preferred disposal routes [25]. Specific legislation enforces minimum recycling limits in some applications. For example, the EU’s End-of-Life Vehicles Directive states that 95% of vehicles must be recycled at their end-of-life [41]. The Resource Conservation and Recovery Act (RCRA) in the US provides a general framework for materials recycling. Alongside this, the 2015 Carbon Fibre Recycling Act estimated a 40% cost saving by using recycled material instead of virgin counterparts [42]. Furthermore, if any waste material is not recycled, there is often a significant cost to landfill it. The cost per tonne varies across EU countries but reaches up to ~EUR 100 (USD 110) in Latvia [43]. The UK has amongst the highest landfill costs at GBP 103 (USD 135) including gate fees [44]. This is also set to rise to GBP 126 (USD 165) in 2025/26 [45]. In the US, landfill tax is set at state level, but on average was USD 57 per tonne in 2023 [46]. Despite these charges, economic analysis has suggested that landfilling and incineration are still the cheapest disposal pathways [47]. However, this analysis did not consider the potential reputational economic cost to FRP producers and users if they are not perceived by their customers to be manufacturing materials in a resource-efficient manner. Although legislation can drive process economics, it will still be necessary to reduce the cost of FRP recycling and/or maximise the value of the recovered material. As described in this section, this may be achieved by realising a fully circular economy for FRPs and finding secondary applications for both the fibre and matrix components. Doing this would also deliver an environmental benefit, as discussed below.

**Table 2:** Products obtained from solvolysis of a thermoset resin described in [48] and associated financial value.

Molecule	Structure	Cost (USD/g)	Reference
2-methoxy-furan	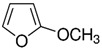	72	[49]
1,2,3-Trimethyl-benzene	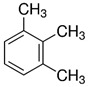	175	[50]
2-methyl-2-hexanol	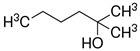	7	[51]
Phenol	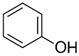	<1	[52]
4-ethyl phenol	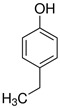	10	[53]

### 2.2. Environmental Impact

During landfill, the economic value and embodied energy of the material is lost. To replace it, it is necessary to manufacture new components, which requires additional resource extraction and energy generation. When waste is incinerated with energy recovery, there may be some displacement of fossil fuels from the electricity grid; however, the emissions associated with this are higher than the combustion of natural gas [10]. It is, therefore, clear that from an environmental perspective, efficient recycling technologies are a much-preferred waste management strategy. By recovering materials and retaining their value, emissions associated with the manufacture of virgin fibres and polymers can be avoided. To quantify these benefits, many life cycle analyses (LCAs) have been conducted considering mechanical recycling [54], pyrolysis [55,56,57], and solvolysis [55,58,59]. The primary environmental performance criteria here centre on energy demand, global warming potential (GWP), human toxicity, ecotoxicity, and fossil-resource depletion. The energy required to manufacture carbon fibres is significantly higher than that needed for glass fibres and resin systems; it has been estimated to be in the range of 171 to 286 MJ/kg [3,25,26], but may be as high as 771 MJ/kg depending on the manufacturing route and temperature [30]. This leads to high greenhouse gas (GHG) emissions of 24.4 to 31.0 kg CO_2_,_eq_ per kg of fibre [30,60] (see Table 1). Glass fibres, in contrast, typically demand 13 to 48 MJ/kg and 2 kg CO_2_,_eq_ per kg of fibre [31,32]. There is a large range of energy requirements and GWP of manufacturing the polymer matrix, but this may also be significant, as illustrated in Table 1.

Although published LCAs tend to follow the established standard ISO 14040, there are significant differences in system boundaries, geographical locations, and approach, which render direct comparisons between recycling technologies impossible. However, within the same studies, where comparisons come down to manufacturing virgin FRPs, all demonstrate a significant environmental benefit. In mechanical grinding of CFRPs, the energy demand was estimated at 2 MJ/kg of fibre [54]; less than 1.1% of the energy needed to manufacturing virgin fibres. However, the generation of single, short filaments means that the material is significantly downgraded and will not be used in similar-value applications. Some size reduction is necessary in pyrolysis and solvolysis processes to fit FRP components into processing equipment; however, both approaches are able to recover long, if not continuous, fibres. In fixed and fluidised bed pyrolysis, the high temperatures needed result in a high energy demand, with estimates ranging from 7.7 to 30 MJ/kg of fibre [56,61]. This may equate to 2 to 4 kg CO_2,eq_ per kg of CFRP, depending on the formulation of the recycled product [56,61]. This is similar to other reports in the literature which reported a GWP of pyrolysis of 1.0 kg CO_2,eq_ per kg of FRP [55]. It is worth noting, however, that this GWP is dependent on the energy mix and it is expected that the increasing adoption of renewable energy technologies will act to drive a reduction in emissions.

For solvolysis, there is an additional environmental impact due to the manufacture of solvents and catalysts; however, the significantly lower temperatures needed may lead to a net energy saving. Compared to virgin fibre production, solvolysis has been estimated to reduce GWP by between 2.5 and 21.9 kg CO_2,eq_ per kg of CFRP [62]. In a supercritical water (SCW) system, the GWP has been estimated at 16.2 kg CO_2,eq_ per kg [55], which is relatively high compared to pyrolysis. However, that study did not provide the conditions used for the SCW process. Conversely, other research has found that it is pyrolysis which is more environmentally harmful, with more than double the GWP and human cancer potential than SCW processes [59]. This is likely due to the optimised system modelled, which included integrated heat and methane recovery. As a comparative analysis, this work does not give absolute values for each recycling process [59], and so a direct comparison to other examples in the literature is not possible. Complete LCAs for solvolysis processes using alternative solvents such as alcohols and acetone have not been completed, although it has been estimated that using these solvents may save 20 to 30% of the energy needed for SCW [58]. This is largely driven by the lower critical point, and lower sensible and latent heats of organic solvents compared to water.

**Figure 2:**
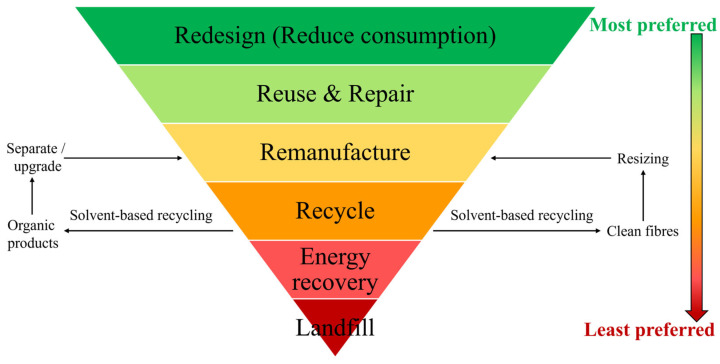
A waste hierarchy for the solvent-based recycling of FRPs, illustrating the most-preferred option (reduce consumption through redesign) to least-preferred (landfilling waste).

In the selection of a solvent-based recycling process, the financial cost, energy intensity and toxicity of the chemicals involved must be balanced against a high yield of both high-quality fibres and organic products. Although it is not clear which recycling technology offers the greatest environmental benefit, all approaches offer an economic opportunity and an improvement in environmental performance compared to the landfilling and/or incinerating of virgin FRPs. As such, the developed waste hierarchy provided in Figure 2 highlights the most- to least-preferred options for FRP waste management, while the following sections review the current state of the art in the solvent-based recycling of FRPs.

## 3. Low Temperature and Pressure (LTP) Processes

Solvolysis may be conducted under low temperature and pressure (LTP, <200 °C, ~1 bar) conditions or at high temperature and pressure (HTP, >200 °C, >1 bar). Compared to pyrolysis, it may be possible to recover more of the original monomers with a chemical recycling process; however, it will be necessary to separate these out from the solvents and/or catalysts used. In addition to strong, clean fibres, the aim of solvolysis is to maximise the recovery of these monomers, or at least, material which can be re-processed into a new polymer. Due to relatively mild temperatures, LTP processes tend to enable the recovery of long chain oligomers from thermoplastics which may be suitable for direct reforming into new polymers. In the case of thermosets, this is often not possible. Most LTP processes rely on the use of strong acids, alkalis or oxidising agents to separate the fibres from the matrix. This frequently generates a liquid mixture which may have significant hazards with regards to human health, safety and the environment. However, it is possible that, with suitable downstream processing, this liquid can be used as a feedstock in new chemical or polymeric products, thus aiding in closing the loop of the FRP life cycle. The necessary process conditions and choice of reaction media are driven by the composition of the matrix material. As this matrix often differs in published examples, the following section aims to provide an overview of different reaction systems and uses selected research as case studies to illustrate developed recycling processes.

### 3.1. Acidic Media

Acidic systems were first considered for the recycling of FRPs in the early 2000s with the application of nitric acid to carbon fibre- and glass fibre-reinforced thermoset resins [63,64]. Here, it was possible to degrade 95% of the resin at just 80 °C; however, long reaction times of 100 h and high acidic concentrations of 4 M were needed. The mechanism for the reaction was determined in later work which found that aromatic rings were nitrated, which subsequently led to the cleavage of C–N bonds present throughout the epoxy network [65]. Similarly, sulfuric acid has also proved capable of recovering clean carbon fibres; however, in this instance, agitation coupled with hydrogen peroxide (H_2_O_2_), a strong oxidizing agent, was also necessary [66]. In the recycling of FRPs, agitation is generally not desirable due to possible entanglement of the fibres. As a result, only non-woven mats may be manufactured as a secondary composite, and they typically have lower mechanical performance than weaves or multi-ply materials. Weak acids, primarily acetic, have also been explored both as a pre-treatment step and as reaction media. In pre-treatment, acetic acid caused swelling of the composite, thus facilitating the mass transfer of H_2_O_2_ and dimethyl formamide (DMF) [67]. As a reactant, an acetic acid/AlCl_3_ system was able to decompose a carbon fibre-reinforced epoxy in 6 h at 180 °C. Here, the AlCl_3_ migrated into the polymer and cleaved the C–N bonds present [68]. Alternative systems include *p*-Toluenesulfonic acid (p-TSA) which, when combined with water and acetic acid, was able to break ester bonds at 180 °C, although relatively long times of 12.5 h were necessary [69].

With regards to fibre-reinforced thermoplastics, the depolymerisation reaction usually proceeds by acid-catalysed hydrolysis. In one example, hydrochloric acid (HCl) recovered both carbon and glass fibres from polyamide-6 (PA6) with the assistance of microwaves. However, it was noted that longer polymer chains and fibre reinforcement necessitated double the concentration of HCl, possibly due to the inhibition of PA6 solvation by the fibres [70]. Other strong acids which have been applied to PA depolymerisation include formic and sulfuric acid, although both were reportedly less effective than HCl [71]. Somewhat surprisingly, weak organic acids at 100 to 120 °C can double the rate of PA depolymerisation compared to dilute HCl solutions when the pH is kept constant. Here, butanoic acid was the most effective, which was attributed to the greater solubility of a long carbon chain into the PA [72]. However, it is worth noting that these experiments used materials that lacked fibre reinforcement, and so conditions may differ when applied to a composite.

### 3.2. Basic Media

Alkaline alcoholysis, glycolysis and aminolysis have all proved effective at degrading a range of fibre-reinforced thermosets. Typically, NaOH or KOH have been supplied as the source of OH- ions. Due to the variety of resins, there is a similarly large range of conditions reported with temperatures of 80 to 200 °C considered along with reaction times of the order of minutes to hours. Again, the conditions are driven by the specific resin formulation and choice of reactant. In one study, it was possible to fully decompose a fibre-reinforced epoxy in a single step using poly(ethylene) glycol (PEG) and 5.6 M NaOH, although relatively high temperatures and long reaction times of 200 °C and 4 h, respectively, were needed [73]. It may also be necessary to use multiple processing steps. For example, in the alkaline degradation of a carbon fibre-reinforced epoxy using a PEG 400/KOH system (160 °C, 200 min), it was only possible to recover clean fibres following a nitric acid pre-treatment and subsequent cleaning with acetone and ultrasound in which 23–30% of the resin was degraded [74]. As an alternative to PEG, mono-ethanolamine (MEA) may be used alongside 0.5 M KOH. This converted an anhydride-crosslinked epoxy into complex amides in just 60 min at 160 °C [75]. This suggests that MEA may be a more effective depolymerisation agent than PEG; however, it is not conclusive as the concentration of KOH was not provided in [74].

Due to the high glass transition temperature, *T_g_*, it is not possible to sufficiently depolymerise high-performance engineering thermoplastics (e.g., poly(ether ether) ketone, PEEK) using alkaline media under LTP conditions. This is due to the lack of mobility of the polymer chains, which means OH- ions are not able to sufficiently penetrate the polymer until the *T_g_* is reached. Hence, much of the available literature reporting the recycling of fibre-reinforced thermoplastics considered the depolymerisation of poly(ethylene) terephthalate (PET). Similar to thermosets, this may be achieved using glycolysis where bis(hydroxyethyl) terephthalate (BHET), a common PET precursor, can be recovered [76]. Alcoholysis may also represent a green engineering approach to recycling fibre-reinforced PET. For example, with the assistance of microwaves, exceptionally fast reaction times of just 5 min resulted in complete depolymerisation at 120 °C. KOH was supplied at 1.25 M in methanol, and so it was necessary to neutralize the solution with an acid before recovering the monomers and glass fibres [77]. Recently, to avoid the generation of potentially hazardous, highly alkaline mixtures, alternative reaction systems have also been reported, as described in the following section.

### 3.3. Other Reaction Systems

**Figure 3:**
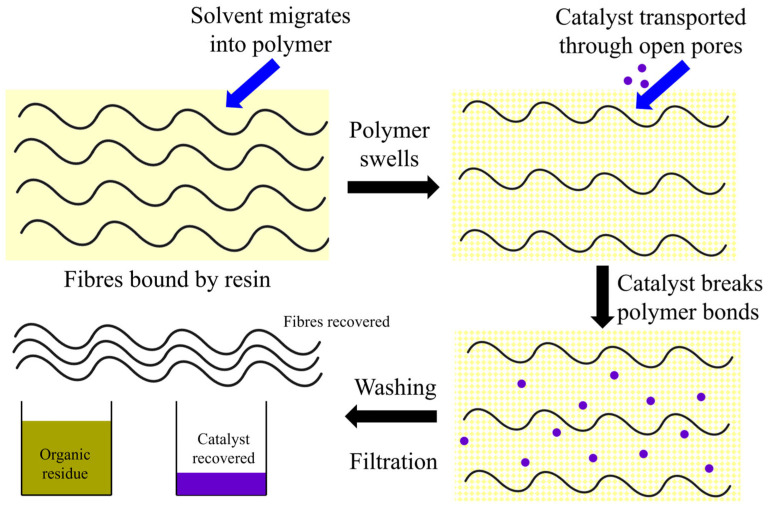
General reaction mechanism for the LTP degradation of FRPs showing the migration of the solvent/catalyst, bond breakage, and recovery of products.

To achieve degradation of a thermoset matrix under LTP conditions without strong acids, alkalis or oxidizing agents, specific catalysts, targeting specific bonds, are needed. For example, diethylenetriamine (DETA) facilitated the rapid degradation of an anhydride-cured epoxy at 130 °C in 50 min due to amination of ester bonds. However, as DETA is a relatively large molecule, a pre-treatment with dichloromethane (DCM) was necessary. The addition of the DCM created pores throughout the resin, without which the DETA could not have penetrated [78]. To avoid the need for this pretreatment, simple transition metal salts (e.g., ZnCl_2_) may be used. These target the amine (C–N) bonds which are frequently present throughout the polymer network. As a relatively small molecule, it can diffuse into a composite, under mild conditions, where the Zn^2+^ ion co-ordinates with, and hence cleaves, amine bonds. This has been demonstrated with aqueous solutions (60 wt.%, 210 °C, 9 h) [79], with ethanol (20 wt.%, 190 °C, 5 h) [80], and with a deep eutectic solvent consisting of thymol and decanoic acid (3.3 wt.%, 180 °C, 1.5 h) [81]. Similarly, other metal salts such as potassium phosphate (K_3_PO_4_) [82,83] and potassium carbonate (K_2_CO_3_) [84] have been explored. To achieve close to complete degradation of the matrix, temperatures of up to 195 °C [82] or times of up to 5 h were needed [84]. However, differences in concentration, solvent, and specific type of epoxy resin mean that it is not possible to fully conclude which reaction system is most effective. It is, therefore, likely that, in designing a commercial FRP recycling process, the exact conditions need to be tailored to the exact resin formulation to be solubilised. To illustrate the process, a general reaction mechanism for the LTP degradation of FRPs is provided in Figure 3.

The same consideration for conditions is also true for thermoplastic-based composites; it does not seem possible to degrade high-performance engineering materials (e.g., PEEK), but fibre-reinforced polyolefins, polyamides, and poly(methyl methacrylate) (PMMA) can be degraded. For example, polypropylene (PP) may be dissolved in xylene at 135 °C whereupon, after 1 h, clean carbon fibres can be recovered. Cooling, acetone addition, filtration, and drying also resulted in up to 93 wt.% PP recovery [85]. A similar process has also been applied to the recycling of glass fibre-reinforced PP [86]. For polyamides, slightly higher temperatures are necessary, which may be attributed to their typically higher glass transition temperatures. One example includes the depolymerisation of polyamide-6 (PA6) which was achieved in a fast reaction time of 1 h, but needed temperatures of up to 160 °C and a benzyl alcohol solvent [87]. Similar conditions are needed when using polar protic solvents such as those investigated in [88], although the exact formulations were not provided. Some commercial resins such as Elium^®^ or Recyclamine contain trigger molecules which enable them to be depolymerised when exposed to certain conditions. Elium^®^, for example, is based on PMMA and may be used in wind turbine components. Glass fibres have been recovered from wind turbine blades following a 72 h immersion in chloroform at room temperature and subsequent methanol treatment [89]. In other studies, a wide range of solvents were investigated and it was apparent that polar solvents such as acetone and ethyl acetate were most effective. Low temperatures from 30 to 70 °C were sufficient to fully degrade the resin; however, under room temperature conditions, long reaction times of 24 h were necessary for the recovery of clean carbon fibres [90]. A selection of different conditions used in LTP recycling processes is provided in Table 3. To avoid the need for strong acids, bases, oxidising agents or other harmful solvents, and to recycle material from high-performance polymers, high temperature and pressure processes, often relying on sub- or supercritical fluids, are often used, as discussed in the following section.

## 4. High Temperature and Pressure (HTP) Processes

### 4.1. Supercritical Fluids

Chemical recycling with sub- and supercritical fluids is a relatively recent (in the past 15 to 20 years) approach, and has already been recognised for recovering fibres from their matrices with virtually no mechanical degradation [96]. Common solvents include water, short-chain alcohols, and acetone. Sub- and supercritical fluids (SCFs) have distinctive thermophysical properties enabling them to achieve efficient and fast resin degradations. The pressure/temperature combination at the critical point alters the intermolecular bonds. In water, for instance, the hydrogen bonds are greatly weakened. This reduces the dielectric constant and enhances solvent–solute molecular action, leading to high water miscibility with all organics. Gas-like high diffusivity, relatively low viscosity, and non-existent surface tension render SCFs excellent reaction media due to their enhanced mass and heat transfer properties, which lead to accelerated reaction rates [97]. Moreover, sub- and SCFs have recently been employed in emerging green processes as they are classed as green reaction media. They are cost-effective, readily available, and they have low potential toxicity [55,98,99]. In addition, sub- and SCFs can be recycled afterward by distillation and can dissolve many organic and inorganic compounds. The following paragraphs give a short review of the main works of FRP depolymerisation in sub- and supercritical fluids.

### 4.2. Solvents and Solvent Mixtures

Similar to LTP processes, the conditions required to recover clean glass or carbon fibres are largely dependent on the matrix type, solvent, and presence of catalysts or other additives. Due to its benign nature and low cost, water has been commonly applied to the recycling of both thermoset- and thermoplastic-based materials. Supercritical conditions (>374 °C, >22.1 MPa [100]) are necessary for chemically stable epoxies [101,102,103]. Here, the low dielectric constant and high miscibility of water with organics allow SCW to effectively break down the polymer matrix while causing no damage to fibres [93]. Additionally, the environmental feasibility of composite recycling was demonstrated using SCW in hydrolysis of a CF/thermoset matrix [104]. Unsaturated polyesters can be degraded in subcritical conditions at less than 300 °C [105]. To enhance the degradation, semi-continuous flow reactors may be used where the solvent is supplied and withdrawn continuously. In such systems, the mass transfer of the degraded organic molecules is likely to be enhanced as the solvent does not become saturated. In turn, this facilitates faster reactions with times as low as 15 min [106]. For the hydrolysis of fibre-reinforced thermoplastics, polyamides are amongst the most widely studied with conditions ranging from 280 °C for 1 h, to 500 °C for 10 min [107,108,109]. These works represent promising systems for a fully closed-loop process due to the recovery of high yields of the monomer ε-caprolactam. However, it should be noted that at high temperatures and long reaction times, secondary reactions (including repolymerisation) can occur [109]. To avoid this, temperatures in the range of 280 to 400 °C appear the most suitable.

As an alternative to water, short-chain alcohols and acetone have also been widely investigated. Although a supercritical state is achieved at a lower temperature and pressure than water, temperatures in excess of 350 °C are often still needed. However, due to the lower pressure, it may be possible to design a lower-cost reaction system than if water was the sole reaction medium. There is a wide variation of resin types considered in the literature and, as polymer chemistry largely determines the reaction conditions needed for degradation, comparison between different solvents is problematic. This is exemplified by two separate works both applying supercritical methanol to the recycling of a fibre-reinforced epoxy. One study achieved complete degradation of bisphenol A (BPA) crosslinked with 1,2-cyclohexane dicarboxylic anhydride at 350 °C in 15 min [110]. However, a commercial epoxy supplied by Cytec (LTM26EL) was only degraded by 60% in the same time, despite a much higher temperature of 450 °C being used [111]. Chemical recycling of CFRPs was also studied using supercritical acetone. Okajima and Sako (2019) found that the decomposition efficiency increased with increasing reaction pressure and acetone density, to a maximum value of 95.6% at 350 °C, 140 bar, and 60 min [112]. Similarly, Sokoli et al. (2017) [113] compared near-critical water and supercritical acetone for the decomposition of composites under a range of temperatures (260–300 °C), pressures (60–300 bar), and composite/solvent ratios (0.29–2.1 g/mL). They found the best conditions for near complete degradation of the resin using supercritical acetone and reported the tensile strength of recovered CF to be retained [113]. This was also observed in work by Okajima et al. (2017) who used sub- and supercritical acetone and various other supercritical solvents (methanol, ethanol, 1-propanol, 1-butanol, 2-butanol, tert-butanol, and methyl ethyl ketone) at 320°C for 6–120 min [114]. Similar conditions are needed for the successful depolymerisation of thermoplastics where both methanol and propanol have been employed. However, it is worth noting that, for PA, methanol caused the further alkylation of ε-caprolactam, reducing the yield to just 14%, compared to the 91% achieved with propanol [115]. A summary of the process conditions needed to degrade various resins with different pure solvents is provided in Table 4.

To further enhance the degradation, or facilitate lower reaction temperatures, solvent mixtures have also been explored [3]. These are typically water combined with short-chain alcohols or acetone. Water molecules attack the ester bonds commonly present throughout a polymer matrix; this hydrolysis reaction is the reverse of esterification. The lower critical point of alcohols and acetone, compared to water, facilitates a reduction in the critical point of the overall mixture and hence enables better diffusivity and massive transfer of the solvents, reactants, catalysts and degradation products. The ratio of water to alcohol or acetone which results in the highest resin degradation likely depends on the polymer type and organic solvent chosen. A selection of examples is provided in Table 4. As an example, a water-ethanol system (1:1 *v*/*v*) achieved a 96% degradation of an epoxy-based CFRP in 15 min at 375 °C [106]. Similarly, a commercial carbon fibre-reinforced RTM6 epoxy resin was fully degraded at 320 °C (120 min) and 340 °C (15 min) using a water–acetone system (1:4 *v*/*v*) [48]. Although solvent mixtures do seem to aid in the reduction of operating conditions, the use of high temperatures (which result in high energy demand, operating costs, and capital expenses) are often still necessary. Hence, recent research has focused on the application of catalysts to further drive down the temperatures and pressures needed in the solvolysis of FRPs, as discussed in the following section.

### 4.3. Catalysed Reaction Systems

Catalysts provide an alternative reaction pathway and may selectively cleave specific bonds within a polymer matrix at lower temperatures than a solvent acting alone. As such, their use also often leads to a reduction in process time. The catalyst choice depends on three key criteria: its solubility in the selected solvent, its lack of chemical reactivity with the solvent and fibre reinforcement, and its ability to attack certain chemical bonds within the polymer. This section aims to provide an overview of the action of these catalysts and draws on examples from the literature to provide specific case studies. The catalysts applicable to the solvolysis of FRPs may be broadly grouped into Brønsted–Lowry bases or weak Lewis acids. Strong acids have not often been investigated under HTP conditions; however, one example is the use of sulfuric (H_2_SO_4_) acid. This can migrate into a thermoset network and hence open it to hydrolytic attack. For example, a 1 M solution reduced both temperature and time from 290 to 260 °C and 75 to 15 min when compared to using water alone [116].

**Figure 4:**
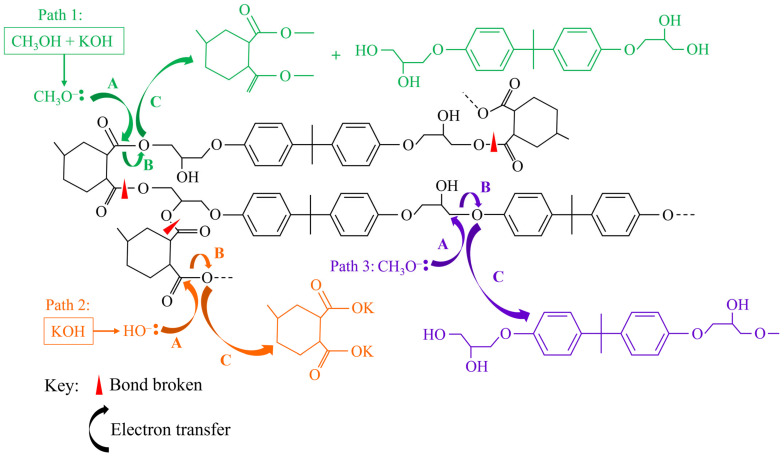
Reaction scheme for the transesterification of an anhydride-cured epoxy resin (adapted from [121]) showing three possible reaction pathways.

Strong Brønsted–Lowry bases which have been considered under HTP conditions include sodium hydroxide (NaOH), potassium hydroxide (KOH), and caesium hydroxide (CsOH). In water alone, OH- ions promote thermal hydrolysis reactions [118], as exemplified by the work of Piñero-Hernanz et al. who degraded > 90% of an epoxy resin with 0.5 M KOH at 400 °C in 15.5 min. Without KOH, only 79% of the resin was decomposed in 30 min at the same temperature [101]. Interestingly, the addition of phenol into the reaction system further accelerates resin degradation. At 315 °C for 30 min, 28% of amine-cured epoxy was degraded using water and KOH, and 15% was degraded using water and phenol. However, 0.18 M of KOH and 1.0 M of phenol dissolved in water enhanced the decomposition to 95%. This phenomenon was due to a free radical mechanism where phenoxy radicals efficiently cleaved C–C, C–N, and ether bonds [119]. Similarly, alkaline hydrolysis has also been applied to the recycling of thermoplastics such as PA6 [120]. In alcohols, KOH may also be used as a transesterification catalyst, following a typical reaction scheme shown in Figure 4. With methanol, for example, an anhydride-cured epoxy was degraded at 220 °C in 120 min with a concentration of just 0.036 M [121]. Amine-cured epoxies generally require higher temperatures to be decomposed, with temperatures of >275 °C used in other work [111]. Here, propan-1-ol with 0.02 M KOH was shown to be an efficient system with KOH, not only forming free radicals, but also promoting the dehydrogenation of propan-1-ol to generate hydrogen. This accelerates resin degradation by hydrogenation and has been shown elsewhere to reduce char formation, leading to improved fibre quality [122].

To avoid the use of strong acids or bases, which may result in complex organic product mixtures which are harmful and/or difficult to dispose of [118], recent attention has been focused on using weak Lewis acids, predominantly metal chlorides. Previous work has applied ZnCl_2_, CuCl_2_, MgCl_2_, and AlCl_3_ to a carbon fibre-reinforced epoxy, all of which facilitate temperature reductions from 330 to 290 °C for a reaction time of 90 min [123]. Interestingly, AlCl_3_ achieved this at a concentration of 0.005 M; an order of magnitude lower than the other additives, thus suggesting that both Al^3+^ and Cl^−^ ions cleave the C–N bonds present [123]; a finding corroborated by other work under LTP conditions [68]. Despite this, most research has focused on the application of ZnCl_2_ which is both less hazardous, and less likely to reduce the mechanical properties of the fibres [124]. In addition to the LTP systems described in Section 3.3 [79,80], a water–ethanol mixture heated to 220 °C required a relatively low concentration of 1.4 M and a time of 5 h to eliminate ~90% of the resin. Here, the enhanced swelling ability of ethanol enabled a lower concentration of ZnCl_2_ to be used [68]. Although these are promising technologies for reducing process temperature, it is necessary to consider the recovery and reuse of these catalytic materials. Although this has been demonstrated at the lab scale [68], it is likely to involve one or more additional process steps which will also impact the economic and environmental performance of a commercial system. Related to this latter point is the need to consider potential metal leaching and the impact of this on regulatory compliance. ZnCl_2_, for example, is amongst the most widely used catalysts; however, Zn^2+^ ions may remain in the pipework. Similarly, chloride ions may remain in solution where they might corrode equipment and/or generate chlorine gas. Therefore, regular maintenance, inspections and gas detection systems are likely needed in order to ensure compliance with relevant health and safety legislation.

The above paragraphs have primarily considered the recycling of thermoset materials due to a lack of literature applying catalysts in the HTP recycling of fibre-reinforced thermoplastics. However, other metal salts, such as caesium carbonate (Cs_2_CO_3_), have been shown to effectively degrade PEEK, a high-performance engineering thermoplastic, frequently used as the matrix for both glass and carbon fibres. It was possible to use very low concentrations of just 0.019 M Cs_2_CO_3_. Here, ethanol–water [125], propanol–water [126], and acetone–water [126] systems were all investigated and, at 350 °C, clean carbon fibres were recovered after a reaction time of 30 min. The presence of water appears to accelerate the reaction, as with alcohol or acetone alone, only negligible phenol concentrations were detected [125,126]. Similar to thermosets, the use of an organic solvent swelled the polymer, created a porous structure and facilitated the mass transfer of the catalyst to active sites within the polymer. Here, Cs_2_CO_3_ may either stabilise the free radicals, prolonging their lifetime, and/or the alkaline nature of the solution promotes the dissociation of protons, thus making them more available to the radicals [125].

Thus far, this paper has set out the environmental and economic case for recycling FRPs, as well as describing solvolysis recycling routes. It is, however, essential to consider the fibres and organic products recovered, which the remainder of this work seeks to do.

## 5. Fibre Properties

In the characterisation of recovered fibres, the primary properties considered are tensile and shear strength, modulus, and surface chemistry, which impacts how well the fibres are likely to adhere to a new polymer matrix. Generally, long fibres are preferred; however, due to size limitations of reactors, it is often necessary to shred a composite prior to removing the resin. To compensate for this, aligned but discontinuous yarns can be manufactured using techniques such as additive manufacturing [127] or the HiPerDif method [128]. Even if only short fibres can be recovered, it is essential that downgrading is minimised by preserving the fibre properties. This section therefore summarises the typical performance of fibres recycled from polymer matrices.

### 5.1. Glass Fibres

Different manufacturers provide differing types of glass fibres. However, they can be broadly categorised into E-glass fibres (higher proportion of Al_2_O_3_ and CaO, lower thermal stability) and T-glass or S-glass fibres (both have higher SiO_2_ content, improved thermal stability and corrosion resistance). The latter type therefore demonstrates greater resistance to degradation during solvolysis of composite materials. As such, only recovered T-glass fibres have been shown to have similar performance to virgin material, while E-glass fibres are generally downcycled into fillers or construction materials [64], although unfortunately, E-glass fibres are much more widely used due to their lower cost [129].

#### 5.1.1. LTP Processes

In LTP processes, the choice of acid has a significant influence on the glass fibre properties. Where 4 M nitric acid, heated to 80 °C, was used, there was negligible mass loss from T-glass fibres while E-glass fibres experienced a mass reduction of 30% [64]. Although not measured, this loss in mass is also likely to reduce mechanical performance. Further research using 6 M nitric acid at 70 °C demonstrated that, compared to virgin material, T-glass fibres experienced only minimal reductions in tensile and shear strength of 3.5% and 2.5%, respectively. In that study, long reaction times of 250 h were necessary. This was decreased by raising the temperature, reaction time, and nitric acid concentration; those measures, however, led to reductions in fibre strength of up to 15% [130], an important consideration for a commercial GFRP recycling process. Acetic acid, combined with AlCl_3_, represents a promising reaction system with regards to maintaining glass fibre properties. After 9 h at 180 °C, the tensile strength was reportedly >96% that of virgin fibres [131]. However, that study did not characterise the fibre surface and so their adhesion to a resin in a new composite material (and hence the likely performance of the composite) is uncertain.

Unfortunately, some reaction systems which are required for the complete degradation of the polymer matrix significantly degrade even T-glass fibres. Sulfuric acid appears to be particularly harmful, with a strength reduction of ~70% observed [132]. The authors noted a loss of the sizing from the fibre surface, which may act to heal surface defects; therefore, were a new sizing to be applied, some of the mechanical performance may be recoverable. Interestingly, not only strong acids may cause fibre degradation. In glycolysis, the tensile strength and modulus was reduced by up to 45% and 55%, respectively, despite clean fibres being recovered at only 130 °C [133]. For these reasons, it may be desirable to explore HTP processes, especially for the recovery of more thermally durable T-glass fibres, as explored below.

#### 5.1.2. HTP Processes

**Figure 5:**
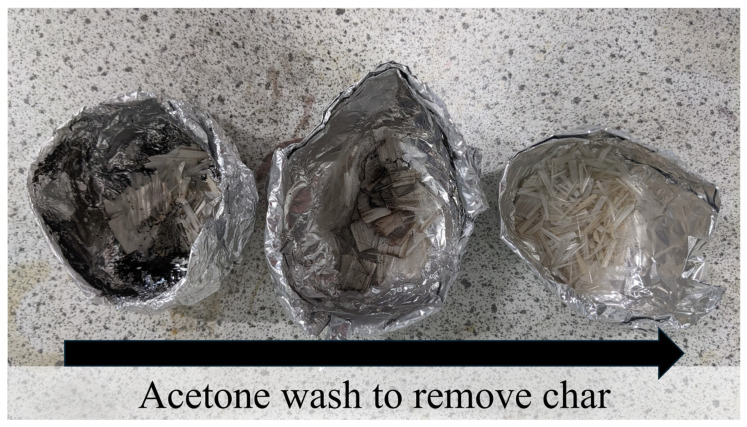
Glass fibres recovered following the HTP process described in [36], showing that clean fibres free of organic residue may be recovered.

The application of organic solvents represents a promising strategy for the recycling of T-glass fibre-reinforced polymers. Images of recovered, clean glass fibres are provided in Figure 5. In supercritical methanolysis with a 4-(dimethylamino)pyridine (DMAP) catalyst at 275 °C, the tensile strength of the recycled fibres was just 6.8% lower than that of virgin material [134]. Similar conditions (260 to 280 °C) were used in a supercritical acetone process and, interestingly, minimal degradation of the less thermally durable E-glass fibres was observed. Here, strength reductions of 11 to 25% were observed [113], suggesting that it is possible to recover E-glass fibres of reasonable quality under HTP conditions. In line with previous work [117,135], increasing reaction temperature resulted in reduced fibre strength. Alternative and less-common solvents also include D-limonene, which is non-toxic, non-hazardous, and often derived from citrus fruits. At 300 and 390 °C, it was possible to recover clean fibres from a polyester resin which were subsequently used to make a new composite. This composite possessed 85% and 64%, respectively, of the strength of a virgin GFRP [136], again demonstrating the influence of temperature on the properties of glass fibres.

Despite being a commonly explored, cheap, and benign reaction medium, water, in contrast to the solvents described above, is particularly detrimental to glass fibre strength at the conditions needed to degrade polymer matrices. Even under subcritical conditions at 280 °C, a reduction in tensile strength of 40% was observed [113], while increases in temperature to 350 °C saw a reduction of up to 60% [117]. Similar effects were seen in both pure water [117] and in the presence of additives such as an oxidizing agent [113] or an alkaline catalyst [135]. The hydronium (H_3_O+) ions generated under these conditions result in metal oxides being leached from the fibre. However, it is worth noting that different oxides will have different reactivity, and therefore the fibre degradation will be dependent on its composition [137]. In terms of glass fibre properties, alkaline environments are possibly the worst choice due to additional degradation chemically etching the fibre surface [135]. In these works, some conditions were identified which resulted in a lower strength loss; however, SEM imaging attributed this to residual resin on the fibre surface [113,135]. This may act as a protective barrier, or seal some defects, although it is likely to affect adhesion to a new matrix and therefore impact the mechanical performance of a secondary composite.

Although some glass fibres may tolerate some solvent-based recycling processes, as illustrated in Table 5, this section has illustrated that they are highly sensitive both to the reaction conditions and composition of the reaction media. As they are usually of lower economic value than the matrix, there is limited research on the properties of these fibres. However, the opposite is true of carbon fibres, hence they are more widely characterised, as discussed in the following section.

### 5.2. Carbon Fibres

Due to exposure to high temperatures during their manufacturing, carbon fibres are generally much more thermally durable than glass fibres. As such, it is generally easier to recover fibres with properties close to their virgin counterparts. However, due to size limitations of processing equipment, recovering long continuous fibres remains a major industrial challenge; often, only short fibres are recoverable due to the shredding of CFRPs prior to a reaction. A further challenge is the removal of a sizing agent from the surface of the carbon fibres during solvolysis. This chemical treatment modifies the surface properties of the fibre and ensures good adhesion to a polymer matrix. Although it generally accounts for less than 2 wt.% of a composite [124], it is essential that this layer is reapplied during the manufacture of a new secondary composite. Despite these challenges, recycled carbon fibres have found applications in non-safety-critical products such as sports equipment, interior aircraft or automotive parts, and laptops [3]. As such, it is vital to consider the properties of the post-solvolysis fibres recovered. Unfortunately, due to differences in fibre type, service life, pre-treatment, and reaction conditions, it is not possible to directly compare different recycling processes. Instead, this section aims to provide an overview of the influence of solvolysis on carbon fibre characteristics.

#### 5.2.1. LTP Processes

Strong acidic [74,138,139] and alkaline systems [74,140] are capable of recovering clean fibres and, in most cases, the tensile strength and stiffness is preserved with properties within 90% of those of fully virgin fibres. Similar to glass fibres, though, there does seem to be a temperature dependence within strong alkaline systems where hotter systems result in weaker fibres [74]. High concentrations of acid also lead to slight reductions in strength; at 15 M, strength retention was measured at 99%, but at 18 M, this had reduced to 94%. Although slight, this was considered outside the range of uncertainty [139]. In addition to tensile properties, interfacial shear strength (IFSS) has also been considered in some work. These tests typically involve curing a drop of resin on the fibre surface, quantifying its dimensions using microscopy, and then measuring the force required to pull that single fibre through the drop. This is highly challenging to achieve in practice and so data for this is not widely published. An example, however, comes from Feng et al., who recovered fibres using an acetone/water system, catalysed by HCl. Their results demonstrated comparable properties to virgin material, suggesting good adherence to a new polymer matrix would be achieved [138]. As an alternative to difficult IFSS measurements, changes in the chemical functional groups have been considered using X-ray photoemission spectroscopy (XPS). In both virgin and recycled fibres, this technique has shown high degrees of both graphitic and amorphous carbon, with major functional groups including carbonyl (C=O), carboxyl (COOH), and single (C–O) bonds. Generally, a higher concentration of oxygen was present on recycled fibres [74,140], possibly due to surface oxidation even at low temperatures in strong acidic or alkaline systems.

More recently, academic research has focused on more benign reaction systems using weak organic acids [95,141], oxidising agents [95,141,142,143], and metal chlorides [68]. All systems can recover clean fibres with minimal deterioration in performance. For example, a very fast process using 1 min of 800 W microwaves, tartaric acid and H_2_O_2_ reported just an 8% reduction in fibre strength. Increasing the process time and proportion of H_2_O_2_ resulted in fibre degradation, reducing strength by up to 23% [141]. These trends are not always as clear. With acetic acid and 20% H_2_O_2_, recycled fibre strength was similar to virgin; however, at a 5% H_2_O_2_ concentration, the strength was reduced by 26% [95]. Increasing the concentration above 20% resulted in an unpredictable change in strength [95]. Alternative oxidising agents include NaOCl [143] and KMnO_4_ [142], and both were observed to cause small reductions in tensile strength of 3.1% and 9.4%, respectively. Interestingly, AlCl_3_ appears to be amongst the least harmful additives when it comes to fibre performance. Although high concentrations of 18 wt.% were used in acetic acid, fibre strength was only reduced by 2.2% [68]. For all of these recycling systems, some surface oxidation was observed which may improve adhesion to a new sizing layer. Although these results demonstrated that carbon fibres can withstand some LTP recycling processes, some may be detrimental to their performance. For example, benzyl alcohol, both neat [87] and with a K_3_PO_4_ [82] additive, caused a 10% to 25% reduction in tensile strength. A second recycling loop also caused a further decrease in stiffness of up to 20% [87]. A summary of the influence of these process conditions on fibre properties is provided in Table 6, and the effects of HTP processes are discussed below.

#### 5.2.2. HTP Processes

**Figure 6:**
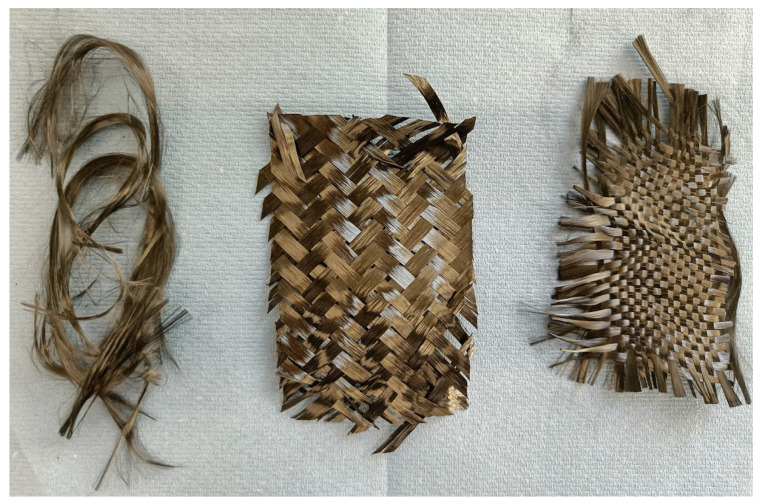
Carbon fibres recovered following the HTP process described in [36] showing that clean fibres, free of organic residue, can be recovered.

Due to wide variations in fibre type, local environment within the composite, lifetime use, and processing conditions, the specific influences of recycling conditions on fibre quality are difficult to quantify. Examples of recovered carbon fibres are provided in Figure 6. However, some generalisations can still be made from the available literature. Although much more thermally durable than glass fibres, carbon fibres sometimes show a similar trend where higher reaction temperatures may result in some reductions in tensile strength [116,144]. However, in other studies, the changes in strength due to high-temperature solvent exposure are more difficult to deduce. Yuyan et al. (2009) reported an average tensile strength of fibres reclaimed in sub-CW to be ~98.2% that of virgin fibres. Also, the fibre surfaces had no cracks or defects and were clean without resin residues [116]. Similarly, Piñero-Hernanz et al. (2008) studied near- and SCW recycling of CFR epoxy composites in a batch reactor. The tensile strength of the reclaimed carbon fibres varied between 90% and 98% compared to virgin fibres [101]. In other aqueous systems [109,119], a negligible difference was reported with even the highest reaction temperature of 400 °C resulting in the highest tensile strength amongst all recovered fibres [109]. However, it is worth noting here that the reaction time was just 15 min, compared to 30 min at lower temperatures. This suggests that fibres may be resistant to changes in their mechanical performance when not exposed to high temperatures for long periods. Supercritical methanol (SC-MeOH) was found to decompose epoxy resin and recover carbon fibres in a semi-batch reactor (250–350 °C, 100 bar, 5–120 min) with fabric shape maintained and no heat damage to the recovered fibres. Okajima et al. (2014) recovered CFs with a strength close to virgin fibres using supercritical methanol (270 °C, 80 bar, 90 min) [110]. Piñero-Hernanz et al. (2008) also used sub- and supercritical alcohols to recover CF, which retained 85–99% of the strength of virgin fibres using batch and semi-continuous-type reactors [111]. In supercritical propanol with KOH, the authors noted that increasing KOH concentrations also resulted in a reduction in fibre strength [144]. In turn, this implies some attack upon the fibres by hydroxyl ions and, from a fibre performance perspective, it may therefore be desirable to use higher temperatures, rather than alkaline catalysts. This trend is not consistent across different reaction systems: KOH and phenol dissolved in water did not cause a statistically significant change in tensile properties [119], thus highlighting the need to characterise the specific fibre types which have been recovered from a specific recycling process. Somewhat unexpectedly, exposure to HTP conditions has also been reported to cause an increase in strength and stiffness compared to virgin fibres, sometimes by as much as 19% [106]. There are two prevailing explanations for this phenomenon. Firstly, residual resin may seal over surface defects which act as stress concentrators and hence become a failure point [111]. In the case where calcination, TGA, or SEM confirm that all resin has been removed, it may be that the recycling process has also removed weaker, graphitic planes from the carbon fibre surface. These planes may contain additional surface defects, hence their removal also removes these potential failure points [145].

The influence of HTP conditions on the surface properties of carbon fibres has also been considered. In the absence of alkaline catalysts, HTP systems generally cause a reduction in surface oxygen concentration and the removal of some functional groups [106,146]. Such fibres are therefore likely to need additional processing before being reused in a secondary composite to ensure good adhesion to a new polymer matrix. In alkaline HTP processes, hydroxyl ions may mitigate this effect by causing some surface oxidation [119,144]. Raman spectroscopy has also been applied to investigate changes in the carbon structure, with results showing that HTP conditions may alter the graphitic planes and cause an increase in disordered crystallinity. These nano-structural changes were also linked to changes in mechanical performance [109]. Atomic force microscopy (AFM) has also occasionally been used to develop 3D images of fibres, and hence analyse their surface roughness. In these works [106,116], little difference was reported between virgin and recycled fibres, which is a promising result; some roughness is likely to be desirable as this may improve adhesion to a new resin. The properties of carbon fibres recycled using a selection of HTP processes are provided in Table 6.

### 5.3. Fibre Resizing

The dominant strategy to mitigate against fibre degradation, and maintain or even enhance the performance of a new composite material, is to apply a new layer of sizing on to the fibre surface. Common sizing formulations typically consist of polypropylene, or polyurethane with an amino-silane coupling agent [147]. As recovered fibres are often non-woven and non-aligned mats, the sizing is usually applied using a batch dip-coating method [147,148], although there have been some reports of the sizing sprayed across the fibre surface [149].

Resizing acts to improve the quality of secondary composites in multiple ways. It may seal over surface defects and hence increase the tensile strength of individual fibres, although some research has reported this had no effect on carbon fibres, and only improved the mechanical performance of individual glass fibres [147]. Resizing binds individual fibres together making them easier to handle and process, reducing both tangling and breakage, particularly for carbon fibres. Finally, it also provides greater adhesion between the fibre reinforcement and a polymer matrix, which in turn enables fabrication of a stronger composite [149]. It is worth noting that the performance improvement due to the resizing may be dependent on the polymer matrix; a carbon fibre-reinforced polyamide did not benefit from resized fibres, whereas an equivalent carbon fibre-reinforced polypropylene composite saw a >50% increase in tensile strength [148].

Overall, solvolysis of FRPs can produce both carbon and glass fibres with a high-quality surface texture, along with minimal loss of shape and tensile strength. Compared to thermal treatments, the preservation of brittleness, length, and orientation is better [58]. Resin residues removed by solvolysis can be recovered ex situ from the solution by evaporation of the solvent, allowing for total recovery of clean, smooth fibres [150]. There is, however, a need to identify suitable secondary markets, predominantly those where lightweighting is useful, but the possible reduction in performance is tolerable. Examples of this include non-structural components of aircraft, automobiles, or sports equipment. To drive confidence amongst OEMs, there is a further need to ensure good quality control of the final product. This is a particular challenge, given the nature of highly mixed and variable feedstocks for any FRP recycling process.

## 6. Organic Products

To maximise resource efficiency and deliver a circular economy for composite materials, it is essential to not only recover high quality fibres, but also identify, upgrade and use the organic products obtained from the resin. These products largely depend on the formulation of the original resin, although they are also influenced by the reactant, solvent, catalyst and process conditions. For thermoplastics, it is possible to recover the original monomers; however, this is mostly not possible with thermoset, crosslinked matrices, as the chemical bonds formed in curing are generally not the same as those broken during degradation. Despite this, it may be possible to recover and reuse these products elsewhere in the chemical industry, as explored in this section.

### 6.1. Products from Thermosets

#### 6.1.1. LTP Processes

Common thermoset resins are based on bisphenol-A epoxies and many LTP recycling systems use an alkaline catalyst. In such a system, and using monoethanolamine (MEA) as the solvent, a mixture of complex amides and organics with hydroxy functional groups were generated [75]. Other work has considered the nitric acid digestion of diglycidyl ether of bisphenol-F (DGEBF) cured with m-phenylenediamine (MPDA) or 1,3-bis(aminomethyl)cyclohexane (BAC) [65]. In the former case, a large mixture of heavy organic compounds was identified, while in the latter, only low-molecular weight compounds were recovered, with picric acid identified as the major product. These examples therefore demonstrate the influence of resin type on organic products.

Metal chlorides have been widely investigated as a suitable additive in LTP processes and, while these systems are able to recover clean fibres, it is apparent that they do not completely break down the matrix. For example, in an ethanol/ZnCl_2_ system applied to N,N′-tetraglycidyl diamino-diphenylmethane (TGDDM) cured with diamino-diphenyl sulfone (DDS), the recovered organic product consisted primarily of oligomers (rather than monomers) with an average molecular weight of 650 Da. Mostly benzene rings and cyclic structures with amine and methyl functional groups were present [80]. Similar results were achieved following the recycling of a diglycidyl ether of bisphenol-A (DGEBA) cured with 3,3′-dimethyl-4,4′-diaminodicyclohexylmethane (DMDC) using acetic acid and 10 wt.% AlCl_3_. Again, benzene derivatives were identified, but the lack of nitrile bonds resulted in a low proportion of amines. The results here also suggested that chlorocarbons were generated and so may need to undergo de-chlorination before being reused [151].

**Figure 7:**
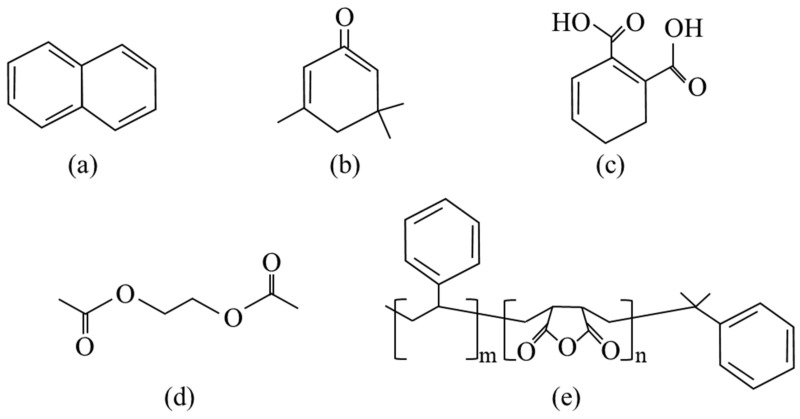
Common organic products recovered after the degradation of unsaturated polyesters, highlighting the likely complexity of the mixture: (**a**) naphtha; (**b**) 3,5,5-trimethylcyclohex-2-en-1-one; (**c**) cis-4-cyclohexene-1,2-dicarboxylic acid; (**d**) ethylene glycol diacetate; (**e**) styrene-co-maleic anhydride oligomer.

With regards to thermoset unsaturated polyesters (UPs), both sulfonic and acetic acids can cleave ester bonds. Here, the product composition consisted of 44 wt.% styrene–maleic anhydride (SMA) copolymer, 33 wt.% ethylene glycol diacetate (EGDA), and 24 wt.% phthalic acid. Although it was not possible to recover a high purity of EGDA, sufficient quantities of SMA were obtained, which may find use as an emulsifier elsewhere in the chemical industry [69]. As expected, for more complex UP formulations, a more complex product mixture might be generated. For example, a resin system consisting of styrene, phthalic anhydride, 1,2-propylene glycol, and cis-butenedioic anhydride, various naphthalene and benzene derivatives, along with esters and amides were identified [152]. For simpler formulations such as vinyl ester crosslinked with N, N-dimethyl-benzenamine (DMA) and dibenzoyl peroxide (DPO), a yield of 80% isophorone was achieved, with the remaining 20% consisting of alkylated aromatics and ketones [153]. The chemical structures of some of these compounds are provided in Figure 7.

Under LTP conditions, it is apparent that thermoset resins are not fully degraded and hence a large mixture of organics are obtained. To ease downstream processing and maximise circularity, it is therefore necessary to develop effective sorting based on resin type and/or close collaborations between material manufacturers, users, and recycling companies to ensure pure waste streams. Where this is not possible, HTP processes may be more suitable due to the further degradation of high-molecular weight compounds into smaller hydrocarbons, as explored below.

#### 6.1.2. HTP Processes

Due to variations in resin formulations, it is not possible to directly compare the organic products recovered using different solvolysis processes. However, some examples from the literature can be used to provide insight into the classes of compounds obtained. In the temperature range 320 to 360 °C, the degradation of a commercial RTM6 epoxy resulted in obtaining primarily alkylated benzene derivatives and short-chain ketones [48]. In later work investigating metal chlorides to reduce reaction temperature, heavier compounds such as adenosine were also detected [123]. Where the resin formulation was known (DGEBA/DDS), similar conditions yielded phenol and amine derivatives [154]. Other research has demonstrated that larger, possibly higher-value, molecules are obtainable. For example, in the degradation of poly(bisphenol A-co-epichlorohydrin) crosslinked with 1,2-cyclohexane dicarboxylic anhydride with methanol, four BPA-type molecules were identified [110]. Similarly, the use of propanol to recycle a BPA/cresol resin recovered a range of light aromatics and medium-molecular weight compounds (220 to 230 g/mol) [111].

Unlike epoxies, which do result in quite a broad range of compounds, the HTP solvolysis of UPs tends to result in higher yields of smaller base chemicals, especially where water is used. Commonly, phthalic acid, benzoic acid, and styrene may be obtained [117,155,156]. Although alcohols often enable a lower reaction temperature, they often cause hydroxy groups of these organic acids to be substituted with short-chain hydrocarbons to form diethyl phthalate and ethyl benzoate, or dipropyl phthalate and propyl benzoate, depending on the alcohol used [157]. These additional reactions may result in the generation of value-added chemicals; however, methods for their efficient separation were not described. A summary of the reaction conditions, resin systems and resultant organic products is provided in Table 7.

**Table 7:** A summary of the organic products detected following the solvolysis of various fibre-reinforced thermosets. Often, products contain cyclic structures which may be useful across the chemical industries; however, the wide range of mixtures will likely result in complex downstream processing.

Polymer Matrix	Solvent/Reactant	Process Conditions	Products	Reference
DGEBA cured with BAC	4 M nitric acid	80 °C	Picric acid Low-molecular weight organics	[65]
TGDDM cured with DDS	20 wt.% ZnCl_2_ in ethanol	200 °C, several hours	Benzene derivatives with methyl/amine groups	[80]
DGEBA cured with DMDC	10 wt.% AlCl_3_ in acetic acid	80 °C, 1.5 h	Benzene derivatives Possible chlorocarbons	[151]
Unsaturated polyester	p-TSA and acetic acid	180 °C, 12 h	Styrene–maleic anhydride (SMA) copolymer Ethylene glycol diacetate (EGDA), Phthalic acid	[69]
Vinyl ester cured with DMA and DPO	Propanol with NaOH or KOH	160 to 240 °C, 60 to 180 min	Isophorone Alklylated aromatics	[153]
RTM6 epoxy	Acetone/water (80:20 *v*/*v*)	320 to 360 °C, 15 to 120 min	Alkylated benzene derivatives Short ketones	[124]
Anhydride-cured BPA	Methanol	270 °C, 90 min	BPA-type compounds	[110]
Unsaturated polyester	Water	380 °C, 5 min	Phthalic acid Styrene Styrene derivatives	[156]
Unsaturated polyester	Water	300 °C, 30 min	Benzoic acid Phenyl acetaldehyde Acetone Ethylene glycol Propylene glycol	[117]
Unsaturated polyester	Benzyl alcohol/K_3_PO_4_	300 °C, 240 min	Benzaldehyde Benzoic acid Phenyl ethyl alcohol Styrene derivatives	[158]
Unsaturated polyester	Ethanol	245 °C, 30 min	Diethyl phthalate Diethyl terephthalate Diethyl fumarate Ethyl benzoate	[157]
Unsaturated polyester	Propanol	265 °C, 30 min	Dipropyl phthalate Dipropyl ester of phthalic acid Dipropyl ester of fumaric acid Propyl benzoate	[157]

### 6.2. Products from Thermoplastics

#### 6.2.1. LTP Processes

Due to the lack of a chemical hardener, it is possible to recover original monomers from fibre-reinforced thermoplastics. However, there is some debate regarding whether this is necessary; if it is possible to recover clean fibres without fully solubilising the polymer, it may be suitable to process oligomers directly into a new polymer. For example, soaking a PA6 resin in benzyl alcohol followed by acetone addition resulted in a PA precipitate [87]. This represents a smaller, less energy-intensive loop within a circular economy framework than fully depolymerising the material. Other work using polar protic solvents enabled recovery of the polymer with only a 10% decrease in its molecular weight [88]. Similarly, degradation of carbon fibre-reinforced polypropylene with xylene facilitated the recovery of up to 93% of the original polymer, although some reduction in the molecular weight was observed [86].

Alternatively, other reaction systems have demonstrated that they are able to recover high monomer yields. In the hydrochloric acid hydrolysis of PA6, adipic acid, hexamethylene diamine, and sebacic acid, amongst others, have all been reported [70]. Weak organic acids perform similarly, with butanoic acid showing the highest hydrolysis rate [72]. Less thermally durable thermoplastics, such as polyesters, may be depolymerised with water, methanol, or ethylene glycol, alongside an alkaline catalyst. Poly(ethylene) terephthalate may be hydrolysed with KOH or NaOH which enables the recovery of the dipotassium or disodium terephthalic acid salt. This salt may then be acidified to regenerate the monomer terephthalic acid (TPA) at a high purity level above 99% [159]. While methanol can also recover TPA as a major product [77], glycolysis yields bis(hydroxyethyl) terephthalate (BHET). Although this is a common precursor in PET production, this process also generates oligomers which may be difficult to separate [76].

Unfortunately, high-performance thermoplastics which are commonly used as FRP matrix materials possess a high glass transition temperature (*T_g_*), and as such, require HTP conditions to degrade. The chemical products obtained from these materials are explored in the following section.

#### 6.2.2. HTP Processes

High-performance thermoplastics frequently used in FRPs include polyamides (PA), polysulfones (PS), and poly-aromatics, such as poly(ether ether) ketone (PEEK). Often, polysulfones are highly resistant to chemical attack and can, therefore, only be mechanically or pyrolytically recycled [160]. For this reason, this section primarily considers the organic products obtained following the HTP recycling of PA and poly-aromatics.

Fibre-reinforced polyamides are both widely used and have a high potential to contribute towards a circular economy. Different solvolysis systems are all capable of recovering high-quality carbon fibres and a high yield of the monomer, ɛ-caprolactam, especially with hydrolysis, as summarised in Table 8. However, this monomer is thermally sensitive and at high temperatures (500 °C) it may be further degraded, or undergo additional reactions to form 1-8-diazacyclotetradecane-2,9,-dione, or even repolymerise [108,109]. Alcohols have also proven effective at degrading PAs; however, where methanol is used, alkylation of ɛ-caprolactam has been reported. Due to the significantly higher activation energy, this reaction was not observed with propanol, with this system recovering up to 91% of ɛ-caprolactam [115]. To maximise resource efficiency, sub- or supercritical water in the temperature range of 300 to 400 °C, or propanol at 370 °C, appear to be the most suitable solvents for the recycling of fibre-reinforced PAs.

Typically, the two major reactants for the synthesis of PEEK are hydroquinone and 4,4′-difluorobenzophenone [161]; however, it does not seem possible to recover these precursors using solvolysis. Instead, the smaller compounds phenol and dibenzofuran are obtained after processing with water–alcohol and water–acetone systems using a Cs_2_CO_3_ catalyst [125]. Phenol, in particular, is an important base chemical, and so, although it has low economic value, it may be useful to maximise its recovery. Here, the dominant reaction mechanism appears to be hydrolysis, as in pure alcohol or acetone systems, the yield of phenol was negligible. Phenolic derivatives (e.g., 4-phenoxyphenol) were also detected in the organic product [125], and therefore, if suitable separation techniques can be developed, it may be possible to obtain some high-value products.

Although not a high-performance thermoplastic, PET is widely used as a matrix, especially for GFRPs. It is also a common consumer plastic and its hydrolysis, alcoholysis, glycolysis and aminolysis have been widely reviewed [162,163,164]. Under HTP conditions, hydrolysis yields both terephthalic acid (TPA) and ethylene glycol; the two main reactants in PET production. As an example, processing at 300 °C provided a TPA yield of 90% at a purity of >97%, indicating that it could be used to produce a new polymer [165]. Methanolysis, however, produces dimethyl terephthalate (DMT) with conversion in excess of 97% observed [166]. For both systems, similar times and temperatures were needed, and so it is important to consider what organic products are desirable, and how easily they can be separated and purified, as discussed in the following section.

**Table 8:** A summary of the organic products obtained following the solvolysis of various fibre-reinforced thermoplastics, illustrating that monomer recovery is possible (e.g., ε-caprolactam), but often, the result is a mixture which will need to be separated.

Polymer Matrix	Solvent/Reactant	Process Conditions	Products	Reference
Polyamide-6	Water	280 to 500 °C, 60 to 10 min	ɛ-caprolactam ɛ-aminocaproic acid	[109]
Polyamide-6	Water	300 to 400 °C, 20 to 35 MPa, 60 to 5 min	ɛ-caprolactam ɛ-aminocaproic acid	[108]
Polyamide-6	Methanol	370 °C, 39 MPa, time not given	ɛ-caprolactam N-methyl caprolactam	[115]
Polyamide-6	Propanol	370 °C, 22 MPa, time not given	ɛ-caprolactam	[115]
PEEK	Water/ethanol (50:50 *v*/*v*), Cs_2_CO_3_ catalyst	350 °C, 30 min	Phenol Dibenzofuran	[125]
PEEK	Water/ethanol (50:50 *v*/*v*), Cs_2_CO_3_ catalyst	350 °C, 30 min	Phenol Dibenzofuran	[125]
PET	Water	200 to 250 °C, 1.4 to 2.0 MPa, 180 to 300 min	Terephthalic acid Ethylene glycol	[76]
PET	Water	250 to 400 °C, 5.0 to 24 MPa, 1 to 30 min	Terephthalic acid Benzoic acid 1,4-dioxane Acetaldehyde Isophthalic acid	[165]
PET	Methanol	280 to 310 °C, 30 to 70 min	Dimethyl terephthalate Ethylene glycol	[166]

### 6.3. Upgrading and Uses of Organic Products

As the plastics industry moves towards using a minimum proportion of non-virgin material in their feedstocks, there is a growing opportunity for organic products from FRP recycling processes to be incorporated into new materials. Ideally, compounds would be reused directly in a new resin. This has been demonstrated by some academic research. However, due to the wide range of polymers, fillers, paints, and other coatings in real industrial waste, some upgrading and hazardous compound removal may first be necessary. As a last resort, the recovered organics could also be used as a fuel; however, this results in a loss of all material value, and so should be discouraged.

#### 6.3.1. Manufacturing New Resins

The recovery of monomers, high-molecular weight oligomers, and even the polymer itself, from fibre-reinforced thermoplastics demonstrates the possibility of achieving close to 100% circularity within the composites industry. In the dissolution of fibre-reinforced polypropylene, for example, the typical oligomer yield was 90 to 93%. After two recycling loops, the manufactured FRP achieved similar mechanical properties to fully virgin material, along with minimal changes in the melting temperature, *T_g_*, or crystallinity [85]. Similarly, in the recycling of glass fibre-reinforced methacrylate, up to 96% of the polymer was precipitated from the reaction solution. Further analysis suggested that this process would be economically viable [89]. In other work, high-performance PA6 has been successfully recovered from composite materials. Although there was a ~40% reduction in composite strength, this was attributed to misalignment of the fibres, rather than the reported degradation of the polymer chains [87]. Although some change in polymer crystallinity was also observed, this research demonstrated the potential for dissolution to achieve a near-circular composites industry. However, it is worth noting here that complete circularity is unlikely to be achieved. Due to the stresses experienced by composites during their service life and the impact of recycling on both fibre and polymer quality, it will almost certainly be necessary to incorporate some virgin material in the manufacture of secondary composites.

Due to the chemistry of thermoset resins, recovery of the monomers seen in the above work on reusing thermoplastic resins is rarely achievable. However, organic compounds can be incorporated into new resin formulations. In one LTP process, recovered organics were blended with virgin material at up to 15 wt.% without a significant reduction in mechanical properties. Increasing the recycled content to 50% did, however, reduce both thermal and mechanical performance [167]. Unfortunately, there is some variation in performance of secondary composites. In another example, using just 10 wt.% of the recovered matrix resulted in a 50% reduction in flexural strength [75]. Alternatively, rather than using thermoset degradation products in a new composite, secondary resins or coatings could be manufactured. A patented process developed by Panasonic Electric Works currently uses the solvolysis of epoxy resin to form glycolic compounds which are used as the feedstock for making polyurethanes [168]. Hydroxylated solvents were also applied to an anhydride-cured epoxy, which resulted in the generation of hydroxylated binders. These were used to make protective coatings with high chemical resistance, scratch hardness, and substrate adhesion [169]. This approach has also been demonstrated elsewhere [170].

Although it is apparent that a high proportion of organic material from FRPs can be reused, this is generally only achieved in practice at small scales. The feasibility of a commercial process will be driven by its economics, and it must be cheaper to use recycled material than virgin. Similarly, the cost of incineration or landfill should also be higher than the developed recycling process. To guarantee a high-quality organic product, it may be possible to separate and upgrade the organic mixture recovered, as discussed below.

#### 6.3.2. Separation and Upgrading Techniques

For the separation of hydrocarbons, distillation remains one of the oldest and most widely used methods across the petrochemical industries. The basic principle relies on the relative boiling points of each fraction; the mixture is heated and more volatile components condense at the top of the column, while heavier molecules condense and fall to the bottom. For simple, often binary, mixtures, a single column may be suitable; however, for more complex mixtures (likely those recovered from HTP processing), a train of multiple columns may be needed [171]. As a separation technique for the purification of polymeric degradation products, it has been more widely applied to pyrolysis systems than solvolysis [172]. However, in the latter approach, distillation is an effective method for removing the solvent; small alcohols such as methanol (boiling point, BP = 65 °C), ethanol (BP = 78 °C), and propanol (BP = 97 °C), as well as acetone (BP = 56 °C), are much more volatile than common degradation products such as phenol (BP = 182 °C) [100]. This is also true for MEA, where distillation at 170 °C recovered the solvent and a mixture of degradation products. At this relatively high temperature, it is possible that some more volatile products were also removed. However, a large proportion of liquid organics were still recovered which were further distilled at 110 °C to remove the moisture present. A new resin was successfully manufactured from this mixture of degradation products [75].

As distillation is an energy-intensive process [172], an alternative could be liquid–liquid extraction where a specific solvent is used to selectively dissolve the organics. The solvent is chosen based on the solubility of the target molecule. For example, methanol can recover high concentrations of phenol [173]. Other common solvents include alcohols, ketones or cyclic compounds, e.g., tetrahydrofuran (THF) [174]. Here, the solvent is mixed with the organic degradation products and left to settle to create a two-phase liquid with the target molecule dissolved in the solvent. This is then removed, and the added solvent extracted via rotary evaporation. An example where this has been successfully applied is in the application of dichloromethane (DCM) to the organics recovered following the solvolysis of a CFRP. Phenolic and aniline-type compounds were extracted after mixing the organic residue with DCM and using successive pH adjustments; phenolics are generally soluble in acids, while aniline and related molecules dissolve in basic conditions [175]. Unfortunately, it is often the case that many similar compounds are generated during solvolysis and that these often have a similar solubility in each solvent. Therefore, liquid–liquid extraction may only be able to separate distinctly different compounds, for example, aliphatics from aromatics, and be unable to obtain a highly pure compound.

The issue of chemically recycling composites is compounded by the likely presence of various contaminants. These may be constituents of the resin (e.g., sulfur from cross-linking agents), filler materials, or coatings. Sulfur represents a particular problem as, if the organics are to be used as a fuel source, strict regulations in multiple countries limit sulfur content. Many different desulfurization methods exist, of which hydrodesulfurization (HDS) is the most common due to its ability to produce a low-sulfur fuel [176]. The organic product is heated to 250 to 500 °C in a gas mixture consisting of hydrogen and nitrogen. In the presence of a zeolite, alumina, metal oxide, or activated carbon catalyst, hydrogen sulfide is generated and subsequently removed from the organics [177]. A removal rate above 99% is possible with this technique [178]. To avoid the large hydrogen consumption and high temperatures, other methods include adsorptive desulfurization (ADS), extractive desulfurization (EDS), and oxidative desulfurization (ODS). These eliminate the need for a fresh hydrogen supply and avoid high temperatures by selectively dissolving sulfurous compounds in solvents such as methanol [179]. Generally, a combination of these techniques is needed to achieve a similar sulfur removal rate to HDS [180]. Where dibenzothiophenes are present, precipitate desulfurization (PDS) may be more suitable where halogenated organics are reacted with the sulfurous aromatics to form insoluble complexes which are removed via filtration [181]. Finally, bio-desulfurization (BDS) uses microbes at ambient conditions to reduce sulfur content. Although this shows selectivity towards certain compounds, it is slow compared to other techniques and remains only at pilot-plant scale [182].

In addition to sulfur, halogenated compounds may also present a risk to the quality of the organic residue. Many waste FRP materials contain chlorine, while brominated compounds are used as a flame retardant [183]. During solvolysis, particularly under HTP conditions, both halide and alkyl radicals may be formed which subsequently generate an acidic gas. This is both hazardous to the local environment and may damage processing equipment [184]. The gasses must therefore be captured as they are generated or treated downstream. The former approach relies on mixing shredded FRPs with a removal agent (e.g., CaO, MgO, Fe_3_O_4_, NaHCO_3_, or Na_2_CO_3_-ZnO), typically at 75 to 100% of the mass of waste [184,185,186]. The halide radical reacts with the metal oxide to create a metal chloride salt. This tends to reduce, not eliminate, halides from the gas phase and so downstream treatment (e.g., hot NaOH-NaX zeolite filters) are also needed. Here, the acidic halide gas adsorbs on to the surface while hydrocarbons pass through [187].

#### 6.3.3. Energy Recovery

As a last resort, the obtained organic material could be used as a fuel. According to the waste hierarchy, this is amongst the least preferred options due to the loss of all material value, although the displacement of virgin fossil fuels may provide an environmental benefit. It is likely that some upgrading steps are needed as described above; however, a high-quality fuel gas was recoverable following the solvolysis of a CFRP [175]. Here, the ethylene glycol/water solvent was heated in a batch reactor to 500 °C to produce syngas. In the absence of a catalyst, the gas yield was 40 wt.%, which was increased to 77 wt.% using a Ru catalyst. Although this reduced the hydrogen content, the higher heating value (HHV) of the recovered gas was 22 MJ/Nm^3^ [175], compared to 34 to 52 MJ/Nm^3^ of typical natural gas [188].

Thus far, this review article has explored solvent-based methods for recycling FRPs, the impact on fibre properties, and the organic products which are recoverable from different thermoplastic and thermoset materials. Although these techniques largely remain at the lab scale, there is some commercial interest in these approaches with several organisations using solvolysis to recycle FRPs, as described in the penultimate section below.

## 7. Commercial Solvent-Based Recycling Processes

Due to intellectual property protection, the exact feedstocks, solvents, catalysts and reaction conditions used for commercial recycling processes are often not disclosed by the operating companies. There are, however, some exceptions, and the publicly available information regarding these processes is summarised below.

Globally, the maximum technology readiness level (TRL) of solvolysis appears to be 6 to 7, meaning that a process has been demonstrated at pilot scale in an operational environment [189]. The US, Korea, and Japan are the global leaders in this space with at least one commercial venture in each country. Adherent Technologies Inc. (ATI) is amongst the most established, with a patented wet chemical treatment termed “Jumbo”, which has been optimised for the recovery of clean carbon fibres [190]. Although ATI announced a plant to process 5 tons of scrap CFRP in 2014 [191], they have since only proposed to license the technology and provide operational support [3]. Since then, other start-ups in the US include Closed Composites LLC and Shocker Composites LLC. The former organisation seems to use a mixture of MnCl_2_ and AlCl_3_ catalysts to cleave bonds in an epoxy matrix, which enables a small proportion to be transferred into a reusable bisphenol A prepolymer [192]. However, as noted previously [189], the long reaction times and essential pretreatment may make this difficult to scale. Meanwhile, Shocker Composites have commercialised an LTP ultrasound-assisted process capable of reusing short carbon fibres from aerospace waste in thermoplastics. By combining recycled fibres with ultra-high-molecular weight polyethylene (and other thermoplastics), they are able to produce high-performance parts via injection moulding [193]. Although this represents a good use for recycled fibres, there remains a need to develop a process capable of recovering long continuous fibres. Further, virgin polymers are used in the manufacture of these parts, hence this technology is not yet fully circular.

Outside the US, eastern Asian companies are dominating the solvolysis of CFRPs. Hitachi Chemical (now owned by Showa Denko) developed a process using benzyl alcohol and K_3_PO_4_ at the 200 L scale. This fully degraded the anhydride-cured epoxy to recover carbon fibres with comparable mechanical performance to virgin, non-woven fibres [194]. Also in Japan, Panasonic Electric Works have developed a hydrolysis process for the recycling of 200 t/yr of GFRP [195]. The recovery of monomers (mainly glycols) and styrene-fumaric acid copolymer are, however, only suitable as low-profile additives and fillers in secondary composites [3]. Elsewhere, Catack-H in South Korea use shredded CFRPs and remove an epoxy resin at ambient conditions using a process reportedly costing only 10% of what incineration would cost. The company only describe the use of a “special solvent” [196], although this is thought to be water-based [189].

These various ventures demonstrate the commercialisation potential for solvolysis systems with both carbon fibres and organic products having further uses. However, key challenges remain regarding the length of fibres (short, non-woven materials are generally no longer suitable for use in high-performance applications) and the separation and reuse of organic products in similar value applications. For GFRP recycling, there remains no commercial solvolysis process. There is a further challenge in the sensitivity of solvolysis to different resin formulations and a lack of studies looking at real, end-of-life mixtures of materials. The major bottleneck in commercialising an FRP solvolysis recycling process seem to centre on the lack of a definitive market for the reclaimed fibres and/or organic products. Due to the wide availability of cheap, virgin material, particularly for glass fibres, there is no significant economic driver for manufacturers to use recycled counterparts. Different processes face different limiting factors in their scalability. For LTP systems, particular consideration needs to be paid to the storage, transport, use, and downstream processing of harmful, eco-toxic materials. In HTP processes, the primary barrier is likely to be the high cost of equipment which is capable of withstanding the high pressures and temperatures used, which likely limits the profitability of a commercial process. Despite these challenges, the relatively high TRL of some of these technologies, particularly in the US and Japan, shows that solvolysis is well-positioned for the recycling of FRPs, provided that suitable markets for both fibrous and organic products can be developed.

## 8. Conclusions

As described herein, solvolysis of FRPs represents a promising route for the recycling and reuse of these materials. This technology offers significant environmental advantages over landfilling and incineration due to the loss of valuable materials and embodied energy which those disposal processes involve. LCAs have shown that solvolysis itself has a lower energy demand and global warming potential (GWP) compared to these traditional disposal methods. Broadly, the process involves depolymerizing or degrading the resinous matrix using a combination of solvents, reactants and catalysts which separates the fibres from the resin. A range of methods have been applied at several different scales and have reached various levels of commercial readiness. Typically, these processes are classified based on their conditions: LTP refers to those which typically operate below 200 °C and atmospheric pressure, while HTP systems are those above 200 °C with an elevated pressure. The choice depends on the resin and fibre type, the target organics to be recovered, and economic considerations. The economic viability of any solvolysis process stems from the recovery of high-value components from FRP waste. For CFRPs, the carbon fibres are the higher-value product, and most processes are optimised for their recovery and maintenance of their mechanical properties. Optimising process conditions to minimise fibre degradation is therefore crucial to ensuring the quality of the recycled fibres. For GFRPs, the focus is on recovering valuable organics. Unsurprisingly, the organic products obtained also vary depending on the resin formulation, solvent, catalyst and process conditions. While thermoplastics do have the potential for recovering oligomers or monomers, thermosets typically yield a complex mixture of compounds. Effective separation and upgrading techniques are therefore needed to maximise the value of these materials. This may include distillation, liquid–liquid extraction, and various desulfurization and dehalogenation methods.

Although this review article has provided a comprehensive overview of solvent-based recycling technologies, there are some limitations to the research presented herein. The literature search relied primarily on Science Direct, Web of Science, and Google Scholar and a systematic approach was not applied. Instead, common keywords in this field were used to return primary research papers and hence provide an overview of the current state of the art. This approach has meant that the resulting review paper is intentionally broad but may not have included some less well-known, or less-cited works. In addition, there are some areas where the research is particularly lacking, particularly regarding the analysis, separation, and upgrading of organic products. For example, dehalogenation techniques have not been applied specifically to the organic products recovered from FRPs, and so this work has relied on drawing information from sources which considered only the solvolysis of common matrix materials, without a fibre reinforcement. Finally, there is little academic literature available detailing commercial solvent-based FRP recycling processes. Although some ventures have been realised, the need to protect intellectual property dictates that specific solvents, catalysts, and process conditions are generally not published. As such, there is little discussion regarding industrial scale processes in this review.

Despite extensive academic research, there is a relative lack of commercialised solvent-based FRP recycling processes. This may be addressed by considering future research into recovering long, continuous fibres, in addition to preserving the fibre properties. Long, high-quality fibres give rise to higher-quality composites, and therefore represent higher economic value, when compared to short-chopped fibres which are frequently used in non-woven mats. Secondly, the separation, recovery, and possible upgrading of organic products remains in its infancy. This is a complex task given the diversity of resin formulations and subsequent range of organic products. As such, further research into a low-cost, energy-efficient separation process is essential, and will also drive the economic case for the solvolytic recycling of FRPs. Commercialisation is further hindered by the wide availability of relatively cheap virgin materials. Regulation supporting the adoption and use of recycled material (such as the introduction of the UK’s Plastic Packaging Tax) would accelerate the development of an economically viable FRP recycling process. Additional research in these areas would help position solvolysis as a key technology for achieving a circular economy for FRPs, reduce their environmental impact, and unlock further economic opportunities.

## Figures and Tables

**Table 1:** Prices, energy demand, and global warming potential (GWP) of common reinforcements, polymers, and metals, showing the relatively high financial and environmental cost of carbon fibres, compared to glass fibres, plastics and metals.

Material	Cost (USD/kg)	Energy Demand (MJ/kg)	GWP (kg CO_2 eq_/kg)	References
Carbon fibre	30–85	171–771	24.4–31.0	[24,26,29]
Glass fibre	0.75–3	13.0–48.3	2.0	[27,28,30,31]
Epoxy	4–20	76–144	4.7–8.1	[26]
Polyester (PE)	1–2.50	63–78	2.8–3.1	[26]
Nylon	2–3	139–145	6.5–8.3	[26]
Polycarbonate (PC)	2.50	80–112	6.0–7.5	[26]
Polypropylene (PP)	1.50	22–112	1.9–2.6	[26]
Polyvinyl chloride (PVC)	1.50	53–80	2.2	[26]
Low density polyethylene	1.50	65–92	1.8	[26]
Aluminium	2.2–3.5	197–298	12	[32,33,34]
Steel	1.6–6.2	25.0–44.6	2.3–2.5	[35,36]
Copper	3.5–5	30–90	1.0–9.0	[37,38,39]

**Table 3:** Summary of low temperature and pressure conditions used to recycle carbon and glass fibres from FRPs (CF = carbon fibre, GF = glass fibre).

FRP	Reaction System	Process Conditions	Ref.
CF-reinforced thermoset epoxy resin	8 M nitric acid	90 °C, 5 h, 40 g_composite_/L_reactant_	[91]
CF-reinforced thermoset epoxy	12 M nitric acid	90 °C, 6 h, 40 g_composite_/L_reactant_, flow reactor at 60 mL/min	[92]
GF-reinforced anhydride-cured epoxy	4 M nitric acid	80 °C, unspecified time	[64]
CF-reinforced anhydride-cured epoxy	Sulfuric acid and H_2_O_2_, concentration unknown	110 °C, several hours with agitation	[66]
CF-reinforced epoxy cured with vinyl ethers	0.1 M HCl and THF/water at 9:1 *v*/*v*	Room temperature, 24 h	[93]
GF-reinforced polyamide	2.5 HCl/amide mol ratio in water	200 °C, 10 min, with microwaves	[70]
CF-reinforced polyamide	2.5 HCl/amide mol ratio in water	200 °C, 10 min, with microwaves	[70]
CF-reinforced amine-cured epoxy	Acetic acid pretreatment, 30% H_2_O_2_, acetone	Acetic acid refluxed at 120 °C, 30 min, acetone wash 120 °C, 30 min in H_2_O_2_ and acetone (1:1 *v*/*v*)	[94]
Aerospace-grade CFRP	14 M acetic acid with 14 M H_2_O_2_	65 °C, 4–5 h, 17 g_composite_/L_reactant_	[95]
CF-reinforced thermoset epoxy	1.4 M AlCl_3_ in acetic acid	180 °C, 6 h, 200 g_composite_/L_reactant_	[68]
CF-reinforced thermoset epoxy	60 wt.% ZnCl_2_ in water	210 °C, 9 h	[79]
Aerospace-grade CFRP	20 wt.% ZnCl_2_ in ethanol	190 °C, 5 h	[80]
CF-reinforced thermoset epoxy	3.3 wt% ZnCl_2_ in thymol/decanoic acid	180 °C, 1.5 h	[81]
GF-reinforced epoxy	0.1 g NaOH/g_composite_ in poly(ethylene) glycol	200 °C, 4 h	[73]
CF-reinforced epoxy	0.1 g NaOH/g_composite_ in poly(ethylene) glycol	200 °C, 4 h	[73]
CF-reinforced anhydride-cured epoxy	0.5 M KOH in mono-ethanolamine (MEA)	160 °C, 60 min	[75]
GF-reinforced PET	1.25 M KOH in methanol	120 °C, 5 min, with microwaves	[77]
CF-reinforced thermoset epoxy	K_3_PO_4_ in benzyl alcohol at 1:10 *w*/*w*	195 °C, 40 min	[82]
GF-reinforced thermoplastic Elium^®^ wind turbine	Chloroform dissolution	72 h	[89]

**Table 4:** Summary of high temperature and pressure conditions used to recycle carbon and glass fibres from FRP with pure solvents and solvent mixtures (CF = carbon fibre, GF = glass fibre).

FRP	Solvent	Process Conditions	Ref.
CF-reinforced anhydride-cured epoxy	Water	440 °C, 35 min, 30 MPa	[99]
CF-reinforced RTM6 epoxy	Water	375 °C, 15 min, 25 MPa, semi-continuous flow reactor	[106]
CF-reinforced amine-cured epoxy	Water	290 °C, 75 min	[116]
GF-reinforced unsaturated polyester	Water	300 °C, 30 min, 0.01 g_resin_/L_solvent_	[117]
CF-reinforced anhydride-cured epoxy	Methanol	270 to 350 °C, 120 to 10 min, 8 to 10 MPa	[110]
CF-reinforced LTM26EL epoxy	Ethanol	450 °C, 15.5 min, 8.0 MPa, 100 g_composite_/L_solvent_	[111]
CF-reinforced LTM26EL epoxy	Propanol	450 °C, 40 min, 25.4 MPa, 100 g_composite_/L_solvent_	[111]
CF-reinforced amine-cured epoxy	Propanol	320 °C, 25 min, 9.0 MPa	[114]
CF-reinforced amine-cured epoxy	Acetone	320 °C, 20 min, 6.0 MPa	[114]
GF-reinforced epoxy (Araldite LY 1564 SP)	Acetone	260 °C, 30 min, 6.0 MPa, up to 210,100 g_composite_/L_solvent_	[113]
CF-reinforced RTM6 epoxy	Ethanol/water (50:50 *v*/*v*)	375 °C, 15 min, semi-continuous flow reactor	[106]
CF-reinforced RTM6 epoxy	Acetone/water (80:20 *v*/*v*)	320 °C, 120 min, 30 g_composite_/L_solvent_	[48]

**Table 5:** Influence of recycling process conditions on glass fibre properties showing that there may be a significant reduction in performance depending on the reactant, time, and temperature applied.

Fibre Type	Recycling Process	Strength Change	Other Properties	Reference
T-glass fibre	6 M nitric acid, 70 °C, 250 h	3.5% reduction	2.5% reduction in shear strength	[130]
T-glass fibre	4 M nitric acid	Not measured	No mass loss	[64]
E-glass fibre	4 M nitric acid	Not measured	30% mass loss	[64]
T-glass fibres	Sulfuric acid	70% reduction	-	[132]
T-glass fibres	Glycolysis, 130 °C	45% reduction	55% reduction in modulus	[133]
E-glass fibres	Water, 280 °C	40% reduction	-	[113]
E-glass fibres	Water, 350 °C	60% reduction	-	[113]
T-glass fibres	Acetic acid and AlCl_3_, 180 °C, 9 h	<4% reduction	-	[131]
T-glass fibres	Methanol and DMAP, 275 °C	<7% reduction	-	[135]
E-glass fibres	Acetone, 260 to 280 °C	11 to 15% reduction	-	[113]

**Table 6:** Influence of recycling process conditions on carbon fibre properties showing that there is usually a small reduction in strength and stiffness.

Fibre Type	Recycling Process	Tensile Properties	Other Properties	Reference
Not given	1.3 M NaOH, 180 °C, 8 h	Strength: 2.4% reduction	No significant changes in surface composition	[140]
Toray T700	Nitric acid, macrogol 400/KOH, 160 °C, 200 min	Strength: 4.4% reduction Stiffness: 3.1% reduction	Increase in surface oxygen and improved wettability	[74]
24k HS	11 to 18 M sulfuric acid, room temperature	Strength: 0.9 to 5.8% reduction Stiffness: 2.6 to 5.0% reduction	-	[139]
Toray T300 3k	0.1 M HCl in acetone/water (9:1 *v*/*v*), room temperature	Strength: 5.1% reduction Stiffness: 2.3% reduction	Increase in surface oxygen. rCFRP had similar shear strength to vCFRP	[138]
Not given	1.4 M AlCl_3_ in acetic acid, 180 °C, 6 h	Strength: 2.2% reduction Stiffness: 1.9% reduction	~10% increase in surface oxygen	[131]
Not given	14 M acetic acid, 9 M H_2_O_2_, 65 °C, 4 h	Strength: 0 to 26% reduction, dependent on acetic/H_2_O_2_ ratio	Additional COH and COOH groups detected	[95]
Hexcel CF	H_2_O_2_/tartaric acid (2:1 *v*/*w*), 1 min microwave irradiation + 30 min soak	Strength: 8% reduction Stiffness: No difference	Increase in surface oxygen concentration	[141]
Synthesised in-house	Benzyl alcohol/K_3_PO_4_ (1:10), 195 °C, 40 min	Strength: <10% reduction	Surface oxygen comparable to virgin	[82]
Toray T700	Water at 280 to 500 °C, 15 to 20 min	Strength: 7 to 18% reduction	Reduction in purity of graphitic structure	[109]
Not given	Water, 1 M phenol, 0.18 M KOH, 315 °C, 9 MPa, 30 min	Strength: Equivalent to virgin	Slight increase in surface oxygen	[119]
Not given	Methanol, 270 °C, 8 MPa, 90 min	Strength: 9% reduction	Retention of weave structure	[110]
Synthesised in-house	Propanol, 0 to 0.36 M KOH, 320 to 360 °C, 30 to 180 min	Strength: 5 to 15% reduction	KOH caused increase in surface oxygen concentration	[144]
Toray T300 3k	Acetone, 320 °C, 1 MPa, 20 min	Strength: Equivalent to virgin	-	[114]
Hexcel 48192	Water/ethanol 50:50 *v*/*v*, 350 °C, 25 MPa	Strength: 9 to 19% increase Stiffness: <7% reduction	Slight decrease in surface oxygen. AFM showed similar roughness	[106]
Toray T700S	Acetone/water (80:20 *v*/*v*), 320 °C, 120 min	Strength: Slight increase Stiffness: Slight decrease	Increase in surface oxygen concentration	[124]
Toray T700S	Acetone/water (80:20 *v*/*v*), 0.05 M ZnCl_2_, 290 °C, 90 min	Strength: Up to 22% increase Stiffness: ~3% decrease	Increase in surface oxygen concentration	[124]
Toray T700S	Acetone/water (80:20 *v*/*v*), 0.005 M AlCl_3_, 290 °C, 90 min	Strength: 10% reduction Stiffness: 23% decrease	Increase in surface oxygen concentration	[124]

## Data Availability

No new data were created or analysed in this study. Data sharing is not applicable to this article.
